# An examination of the influence of drained peatlands on regional stream water chemistry

**DOI:** 10.1007/s10750-023-05188-5

**Published:** 2023-03-28

**Authors:** Catharine Pschenyckyj, Thomas Donahue, Mary Kelly-Quinn, Connie O’Driscoll, Florence Renou-Wilson

**Affiliations:** 1 School of Biology & Environmental Science, University College Dublin, Belfield, Dublin 4, Ireland; 2 Hanley Ryan, Galway, Ireland

**Keywords:** Peatland, Raised bog, Drainage, Extraction, Water quality, Ammonia, DOC

## Abstract

Currently, 50% of Irish rivers do not meet water quality standards, with many declining due to numerous pressures, including peatland degradation. This study examines stream water quality in the Irish midlands, a region where raised bogs have been all historically disturbed to various extent and the majority drained for industrial or domestic peat extraction. For the first time, we provide in-depth analysis of stream water chemistry within a heavily modified bog landscape. Small streams from degraded bogs exhibited greater levels of pollutants, in particular: total dissolved nitrogen (0.48 mg/l) and sulphate (18.49 mg/l) as well as higher electrical conductivity (mean: 334 μS/cm) compared to similar bog streams in near-natural bogs. Except for site-specific nitrogen pollution in certain streams surrounding degraded peatlands, the chemical composition of the receiving streams did not significantly differ between near-natural and degraded sites, reflecting the spatio-temporal scales of disturbance in this complex peat-scape. Dissolved organic carbon concentrations in all the receiving streams were high (27.2 mg/l) compared to other Irish streams, even within other peatland catchments. The region is experiencing overall a widespread loss of fluvial nitrogen and carbon calling for (a) the development of management instruments at site-level (water treatment) and landscape-level (rewetting) to assist with meeting water quality standards in the region, and (b) the routine monitoring of water chemistry as part of current and future peatland management activities.

**Supplementary Information:**

The online version contains supplementary material available at 10.1007/s10750-023-05188-5.

## Introduction

Peatlands cover 3% of the global land area, are distributed across more than 180 countries, and have been identified as globally significant ecosystems (Yu et al., 2010). Peatlands are carbon sinks and biodiversity hotspots; they store, purify and discharge water; and support many other socio-economic and cultural services (Bonn et al., 2016). Peatlands are the most carbon-dense terrestrial ecosystems on the planet and account for *c.* 10% of the world’s freshwater resources (Holden, 2005). An excess of moisture is key to a healthy peatland and therefore the most critical impacts on peatlands are associated with drainage and changes in their water regime (Lindsay et al., 2014). Peatland drainage has been identified as a primary driver of environmental degradation with a cascade of impacts: greenhouse gas emissions, biodiversity loss, increased fire frequency, land degradation and not least, increased carbon loss and nutrient leaching via water (Biancalani & Avagyan, 2014). With 25% of European peatlands already degraded, new land-use change policies under Common Agriculture Policy (CAP) reform (Anon, 2020; Tanneberger et al., 2020) may see a reduction in drainage and the implementation of rewetting of drained peat soils.

**Fig. 1:**
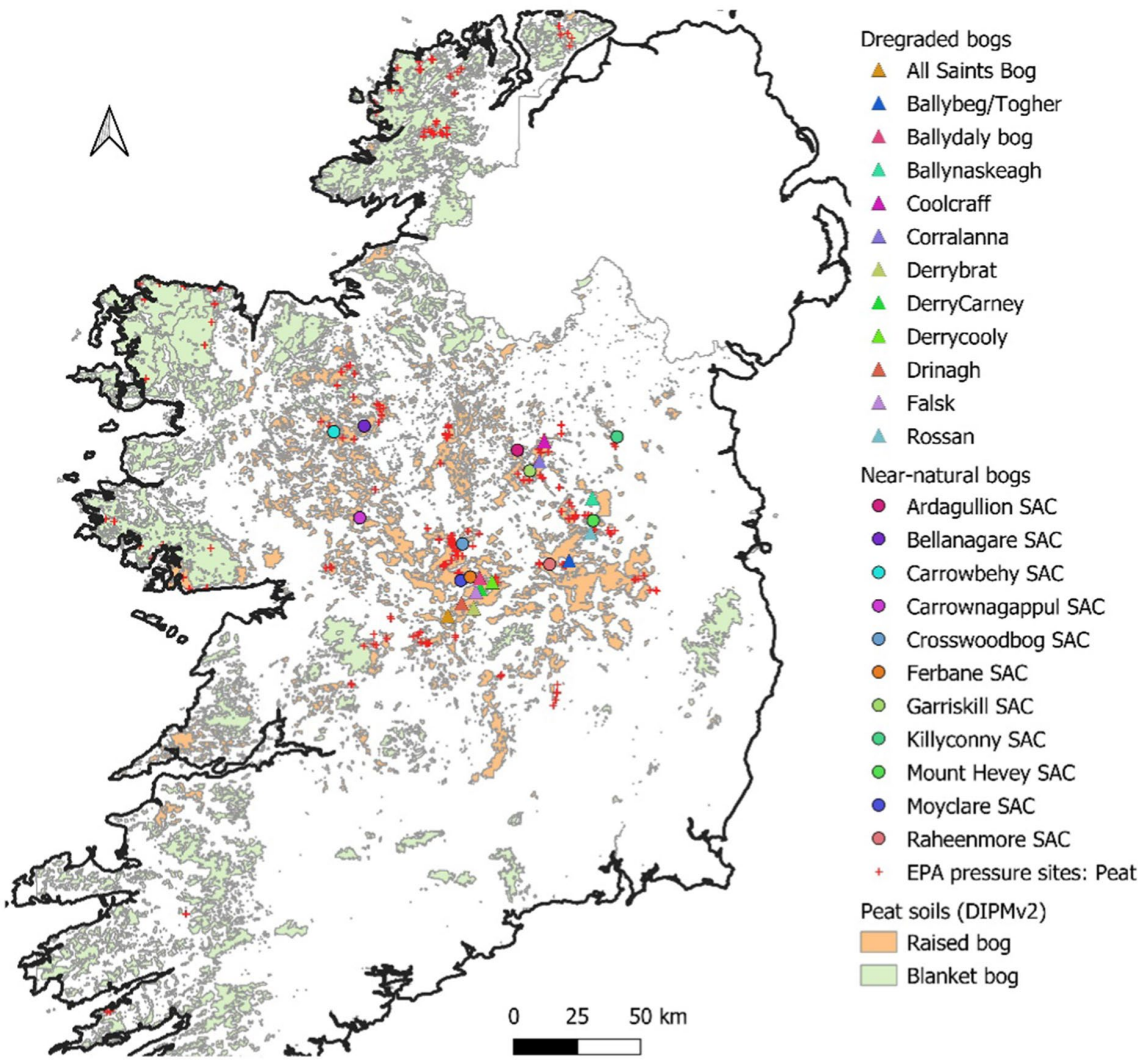
Map of Ireland showing peatlands types (Connolly & Holden, 2009), Environmental Protection Agency pressure sites (red dots, EPA, 2020b) associated with peat and locations of degraded (triangles) and near-natural (circles) bog sites sampled in this study

The variety of peatland types found in Ireland (mainly raised and blanket bogs with a small area of fens, Fig. 1) is a function of their water supply (Heathwaite & Göttlich, 1990). While covering 21% of the land surface (Tanneberger et al., 2017), no fully natural peatlands (true mire) remain in Ireland as they have been extensively modified by human activities (Renou-Wilson, 2018). Centuries of domestic turf cutting, as well as decades of industrial extraction and intensive reclamation for agriculture, forestry and infrastructure (windfarms and waste grounds), have led to degraded peat soils, habitats and landscapes (Renou-Wilson et al., 2022). With the absence of pristine mires, the least damaged, near-natural peatlands, which are considered to be of conservation status, have been designated as Special Areas of Conservation (SAC) and cover a mere 18% of the total peatland area in the country (NPWS, 2015). While most of the SAC raised bogs have been subject to various scales of restorative management practices, considerably ramped up since 2018 (NPWS, 2017; DHLGH, 2022), damaging activities and on-going degradation continue across the whole peatland resource (NPWS, 2019), which calls for a new way to manage peatlands in order to return the wide range of ecosystem services that they provide in their natural state (Renou-Wilson et al., 2019).

The potential effects on water-related ecosystem services are intrinsically linked (Flynn et al., 2021; Pschenyckyj et al., 2021), with the relationship between peatlands and water quality being internationally recognised (Rothwell et al., 2010; Nieminen et al., 2021; Williamson et al., 2021; Pickard et al., 2022). According to the latest *Water Quality in Ireland* report, just over half of surface water bodies in the country meet satisfactory water quality levels, with quality continuing to decline (O’Boyle et al., 2019). Poor inland water quality has been attributed to many pressures: agriculture (53% of water bodies at risk), hydromorphology (24%), urban wastewater (20%), forestry (16%), with ‘peat’ (7.5%) ranked a higher pressure than industry (6.5%) (EPA, 2020b). The 2018–2021 River Basin Management Plan identified a total of 119 waterbodies at risk of not meeting water quality objectives in which peatland extraction solely (i.e. excluding forestry and farming on peat soils) was the main contributing factor (DHPLG, 2018).

Peatlands are inherently heterogeneous in space and time (Renou-Wilson et al., 2022) but all interact with the wider landscape via hydrological pathways. In many cases, drainage for peat utilisation has necessitated that the original bog habitat be drained and hydrophytic vegetation be removed, thus lowering the regional groundwater table (NPWS, 2017) and bringing about changes in downstream water quality (Pschenyckyj et al., 2021). Peat and soil water chemistry, hydrology and biological functioning (including downstream biota) are all impacted by drainage and removal of vegetation during peat extraction (Clément et al., 2009; Marttila & Klove, 2010; Heikkinen et al., 2018). Peatland drainage and extraction provide several serious challenges to aquatic life, including increased mortality due to habitat alterations, behavioural changes, as well as changes in community structure and overall reduced species richness (Ramchunder et al., 2009; Donahue et al., 2022).

The application of compulsory water protection guidelines by peat extraction companies vary widely across the globe and their efficiency has been widely questioned given the exceedance levels in pollutants and the lower fish abundance in affected streams (St-Hilaire et al., 2006; Pavey et al., 2007; Clément et al., 2009). Several Fenno-Scandinavian studies point to more successful mitigation measures for reducing nutrient and sediment loads, such as overland flow areas (that purify the water by means of natural bio-physical and chemical processes), when used in conjunction with other water pollution control methods, such peat lifting practices, field ditch retainers and sedimentation basins (Heikkinen et al., 1995; Huttunen et al., 1996; Marttila and Kløve 2009). 22% of all Irish peatlands is affected by peat extraction (Renou-Wilson et al., 2022), and while water pollution is acknowledged as the most significant risk from such operations (Ministry of the Environment 2013), there is a lack of research focusing on the extent and efficiency of Irish guidelines (EPA 1996; EPA, 2020a) in reducing such impacts. While peat extraction for fuel is now limited to domestic activities, horticulture peat extraction is likely to persist due to the increased global demand (Joosten et al., 2017).

Rehabilitation plans on specific industrial peat extraction sites, either for post or on-going licensing extraction, have not been fully implemented and are limited in their effects (EPA, 2020a). Moreover, rewetting and restoration plans for large areas of drained peatlands have been proposed to help meet climate change mitigation targets in both Ireland and other peat-rich countries (DECC 2020; Ziegler et al., 2021). Peatland restoration has been highlighted as a tool to meet different regulatory targets, not least in relation to the Framework Directive (2000/60/EC, WDF) and its target to achieve ‘at least good status’ in European water bodies (Martin-Ortega et al., 2014). Similarly, reporting of habitat conditions under Article 17 of the EU Habitats Directive (Council Directive 92/43/EEC) (EEC 1992) has continually demonstrated the poor ecological status of European bogs and the urgent need to restore these ecosystems (NPWS, 2019). One of the aims of the restoration programme of designated SAC raised and blanket bogs is to restore the water quality status of catchments, which is significant for both biodiversity and drinking water supply (Flynn et al., 2021). Rewetting and restoration have demonstrated variable results in terms of restoring water quality by decreasing the concentration of suspended solids and dissolved organic carbon (DOC) in the peatland water with positive consequences for meeting drinking water requirements (Wallage et al., 2006; Armstrong et al., 2010). More specifically, rewetting of peat-extracted bogs has been shown to lead to a decrease in DOC by between 36−53% (Glatzel et al., 2003; Waddington et al., 2008). However, the same intervention on cutover peatlands, especially blanket bogs, can be hampered by other factors, for example, historical drainage loss and peat subsidence leading to risk of inundation (Williamson et al., 2017). Studies in Fenno-Scandinavian boreal peatlands systems have found that peatland rewetting can result in reduced concentrations of various pollutants, including inorganic nitrogen (N), base cations and DOC (Lundin et al., 2017; Menberu et al., 2017). Restoration also appears to have the potential to reduce leaching of dissolved organic nitrogen (DON) to downstream waters in the short term (Edokpa et al., 2017a). However, sediments, nutrients and fluvial carbon concentrations that leach from degraded peatlands have been found to vary depending on site-specific characteristics and management, thereby affecting the targets for water quality following restoration/management actions (Gaffney et al., 2018a). The lack of information concerning the current water quality of streams directly influenced by both natural and drained peatlands limits our understanding of the full impacts of their restoration.

In this paper, we provide a snapshot of the quality of streams in one of the European regions most influenced by peatland drainage for peat extraction. In addition to presenting a comprehensive review of the water quality of freshwater systems in the Irish midlands, we hypothesise that significant differences would occur between the chemical components of streams within degraded and natural bogs, and between the upstream and downstream waters that receive these bog streams. As the quality of many water bodies continue to degrade in Ireland (O’Boyle et al., 2019), it is crucial to fully understand the pressures that underpin this trend. While focused on Irish peat catchments, this study will also be informative for other countries with similar issues with peat soil utilisation. Furthermore, in the context of global environmental change, it is important to elucidate the contribution of human activities, such as peat extraction, on the export of pollutants into freshwater systems and the need for more developed site as well as landscape water protection measures.

## Methods

A range of streams were sampled from 12 degraded bogs and 11 natural bogs across the Irish midlands, home to oceanic raised bogs (Connolly & Holden, 2009) (Fig. 1). Of note, all Midlands bogs are remnants from a small number of originally very large bogs (e.g. Bog of Allen) that would have covered most of the centre of Ireland, having developed from a few large lakes that formed in the aftermath of the melting of the glaciers (Feehan et al., 2008). Land use change and management have left remnants of these landforms as individual bogs. Therefore, a bog site may display more than one sub-catchment with specific characteristics (e.g. variable drainage intensity or rewetting activities). In this study, streams were chosen on the basis of these management-specific sub-catchments: 58% of the streams were order 1 & 2 streams with the remainder being order 3 & 4 (these were associated with degraded bogs located close to large rivers) (Online Resource 1). The cluster of streams (within bog streams, and receiving streams upstream and downstream of bog streams) belong to a unique sub-catchment that were on average 30 km^2^ (range: 11 to 109 km^2^), spread over 10 sub-catchments that have all been assessed by the Irish Environmental Protection Agency (EPA) as being ‘at risk of not achieving good status due to ‘extractive industry pressure associated with peat’ or ‘peat related activities’ under the Water Framework Directive (WFD 2000/60/EC) Significant Pressures (EPA, 2020b).

The degraded bogs in our study were either under extraction or had just ceased industrial extraction (fuel or horticultural peat) with additional domestic turf cutting on the margins. The least degraded, near-natural bogs (hereafter natural bogs) were all part of the Natura 2000 network of raised bogs SACs and have been the subject of a range of restorative management practices over various periods, starting 15 years ago but mostly within the last 4 years (Online Resource 1).

**Fig. 2:**
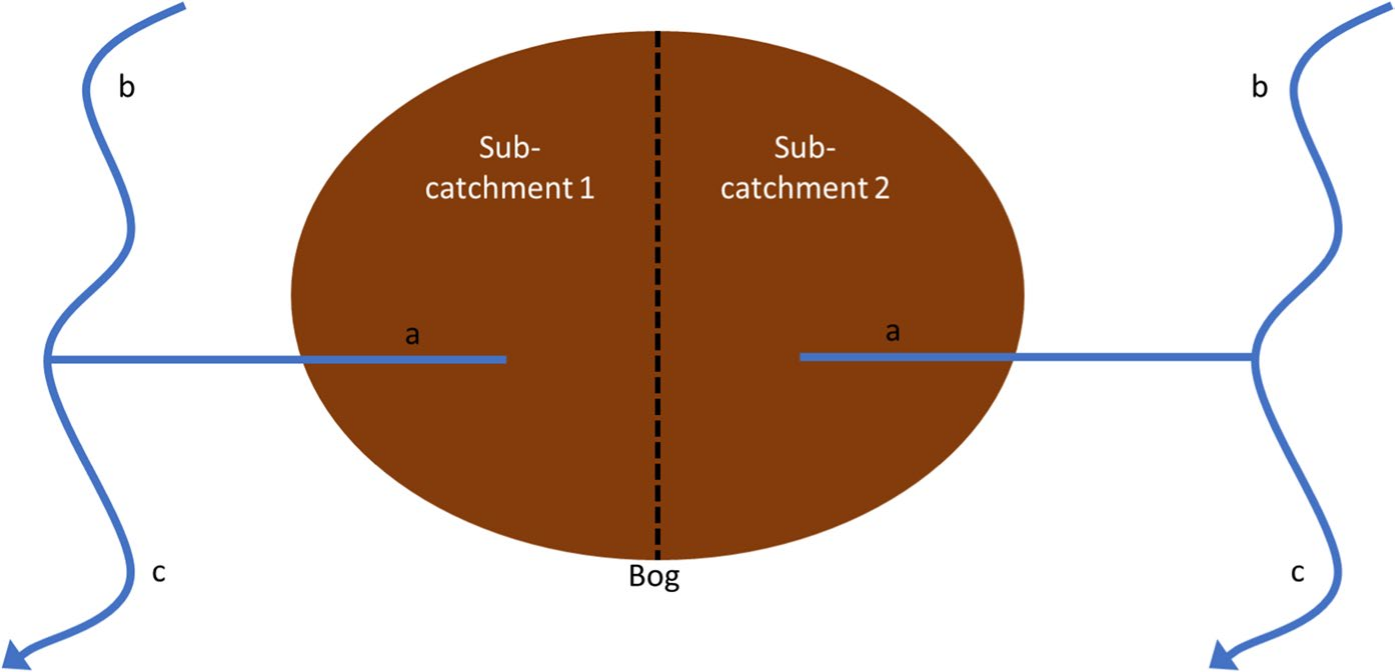
Conceptual diagram illustrating the sampling location strategy in a bog site that contains two sub-catchments, whereby samples were taken from **a** within a small stream in the bog (*within bog SS*), and **b** upstream and **c** downstream receiving streams (*RS*) where the *within bog SS* was a tributary

Sites were selected based on the following criteria: (1) they contain a recognised small stream (SS) that originates in the bog (hereafter *within bog SS*_*,*_ defined further as *within bog SS*_nat_ for natural sites and *within bog SS*_deg_ for degraded sites), and/or (2) contain a receiving stream (RS) or river that flows near the bog whereby the *within bog SS* was a tributary (referred to as ‘*upstream RS’* or ‘*downstream RS’* sampling points), all within one sub-catchment (Fig. 2). Once a waterbody was identified, a sampling location was marked and was GPS recorded (one sample per waterbody). Sampling of RS took place at a sufficient distance to take account of the need for the tributary water to be fully mixed with the receiving stream water (which depended on the size of the receiving stream). All site sampling locations were located 40−80 km away from the coast (Fig. 1) with reduced maritime influence on precipitation, as shown by previous work (Beltman et al., 1993). In total, 63 sites were sampled in each of the three seasons (some sites were dry on some sampling occasions).

Sampling campaigns took place during autumn 2020, and spring and autumn 2021, each over a 3-week period. Samples were kept in cool boxes with ice packs during travel, and in a refrigerator until the start of processing (within 24 h). Methods of analysis for each environmental variable are given in Table 1. All analyses were conducted in either University College Dublin or at an external laboratory that subscribes to the Aquacheck International Testing scheme for Chemical Analytical Laboratories.

**Table 1:** Summary of water quality analyses undertaken, abbreviations and methods used

Water quality parameter	Abbreviation	Analysis method
pH	pH	Measurements taken in the field on unfiltered sample using a portable probe (Hanna Instruments Ltd)
Electrical conductivity	EC	
Total dissolved solids	TDS	
Total carbon, total inorganic carbon, dissolved organic carbon	TC, TIC, DOC	TN/TC analyser (Shimadzu Europa GmbH). DOC calculated as TC-TIC^a^
Total dissolved nitrogen	TDN	
Specific ultraviolet light absorbance at 254 nm	SUVA_254_	Absorbance was measured at 254 nm on samples diluted to < 1 au, using a 1 cm pathlength quartz cuvette. The pathlength was converted from cm to m (multiplying by 100) and SUVA_254_ was calculated by dividing the absorbance value at 254 nm by the DOC concentration in mg/l^a^
Chloride (Cl^−^)	Cl	Ion chromatography (Dionex, Thermo Fisher Scientific Inc.)
Nitrate (NO_3_^—^N)	NO_3_–N	
Sulphate (SO_4_^2−^)	SO_4_	
Sodium (Na^+^)	Na	
Magnesium (Mg^2+^)	Mg	
Calcium (Ca^2+^)	Ca	
Iron	Fe	
Potassium	K	
Nitrite	NO_2_–N	External laboratory analysis^a^
Total Ammonia (TAN as N)	TAN	External laboratory analysis^a^
Unionised ammonia	NH_3_–N	Calculated (FDEP 2001)
Orthophosphate	OP	External laboratory analysis^a^
Turbidity	Turbidity	External laboratory analysis
Biological oxygen demand	BOD	5-day incubation period. External laboratory analysis
Total dissolved phosphorus	TDP	Sulphuric digestion and colorimetric determination Murphy and Riley (1962)^a^
Dissolved inorganic nitrogen and dissolved organic nitrogen	DIN, DON	Through calculation: DIN = NO_4_–N + NO_2_–N + NH_4_–N DON = TDN-DIN

^a^Analysis performed on samples filtered to 0.45 μm are identified

### Data analysis

All data analysis were performed in R statistical package (R Core Team, 2019). Relationships between water quality parameters were assessed using a Spearman’s Rank test, as well as to examine the strength and direction of the relationship (*r* value). To avoid multicollinearity from the ANOVA analysis, the variables which showed strong collinearity (based *r* values greater than 0.7) were removed from the analysis. This was largely based on removing one variable per correlated pair, but in some instances we kept only one variable that was most centroid from the cluster following a principal component analysis (PCA) (Dormann et al., 2013). A multivariate PCA approach was used for explanatory data analysis to visualise variation and multicollinearity in the data. Two PCAs were performed: one to compare *within bog SS*_nat_ and *within bog SS*_deg_ water quality (PCA1), and the second to compare the water quality of downstream *RS* (from degraded and natural bogs) (PCA2) (Online Resource 7). Bartlett’s (*b* ≤ 0.05) and Kaiser–Mayer–Olkin (KMO ≥ 0.5) tests were used to check the appropriateness of the PCAs (Field et al., 2012).

Prior to performing ANOVA tests, the assumptions of ANOVA were checked and data transformed where necessary (see Online Resource 3–7). Type II ANOVA was used to account for the unbalanced design of the study (Langsrud , 2003; Smith & Cribbie, 2022). The Bonferonni Correction was applied to ANOVA results to correct for multiple comparisons inflating Type 1 errors.

Various ANOVA tests were performed to assess (1) differences between seasons for *within bog SS* (ANOVA 1a) and *RS* (ANOVA 1b) data; (2) differences between degraded and natural sites for *within bog SS* (ANOVA 2); and (3) differences between upstream and downstream *RS* for both degraded (ANOVA 3a) and natural (ANOVA 3b) bogs (see Online Resource 2). Sampling period was included as a factor in ANOVA 2 in order to assess seasonal influence on the significance of results between groups. Where significant differences occurred (*p* value < 0.05), a post-hoc test was performed using ‘HSD.test’ function to assess whether significant differences occurred between specific groups.

## Results

### Correlation between variables

**Fig. 3:**
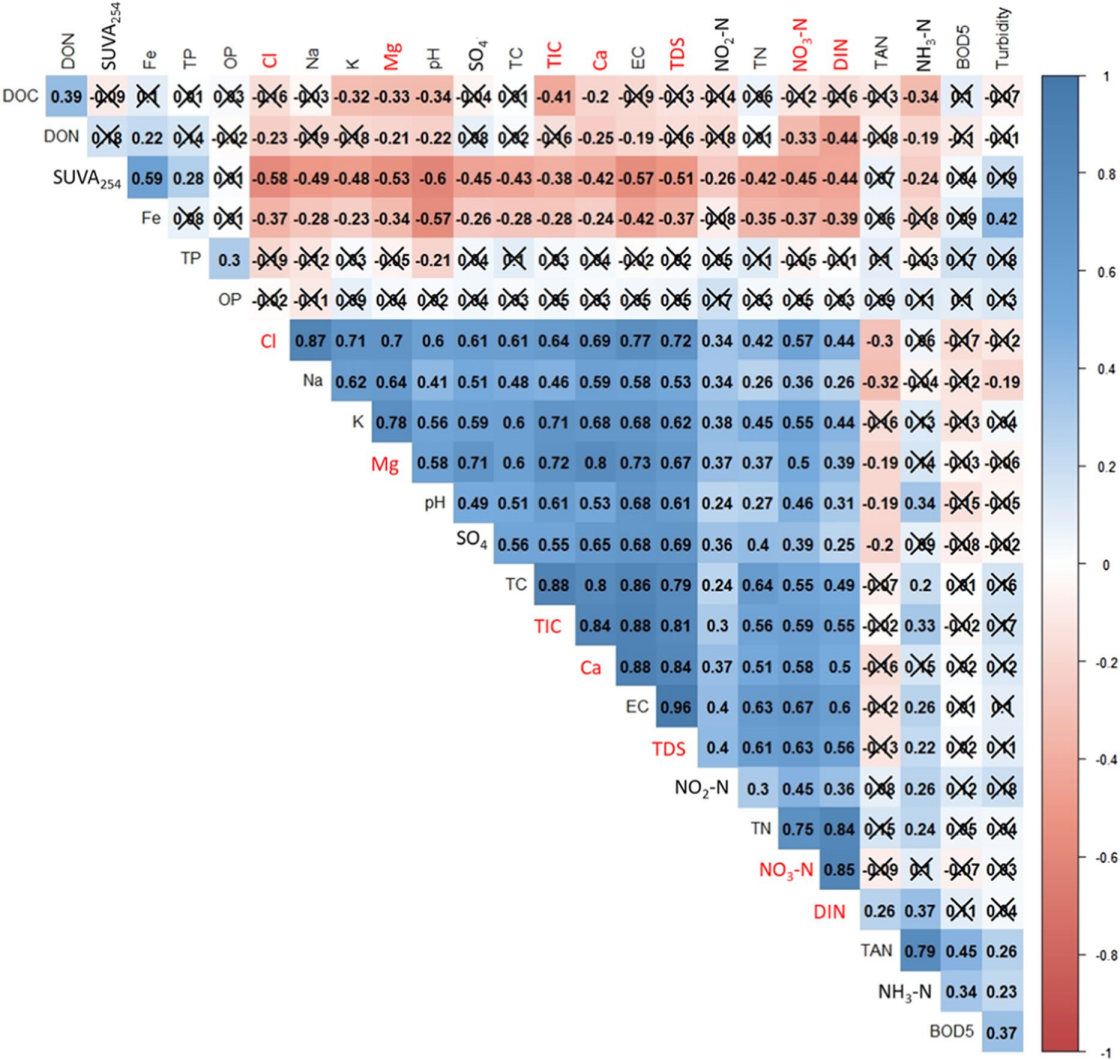
Correlation matrix based on the results of Spearman’s Rank correlation tests that compared water quality variables. Direction of correlation is indicated by colour (red indicates a negative relationship, and blue a positive relationship). The depth of colour and r values (− 1 to + 1) indicate the strength of the relationship. Crossed out boxes indicate an insignificant relationship (*P* ≥ 0.05). Variables in red were removed from subsequent ANOVA analysis due to multicollinearity

**Fig. 4:**
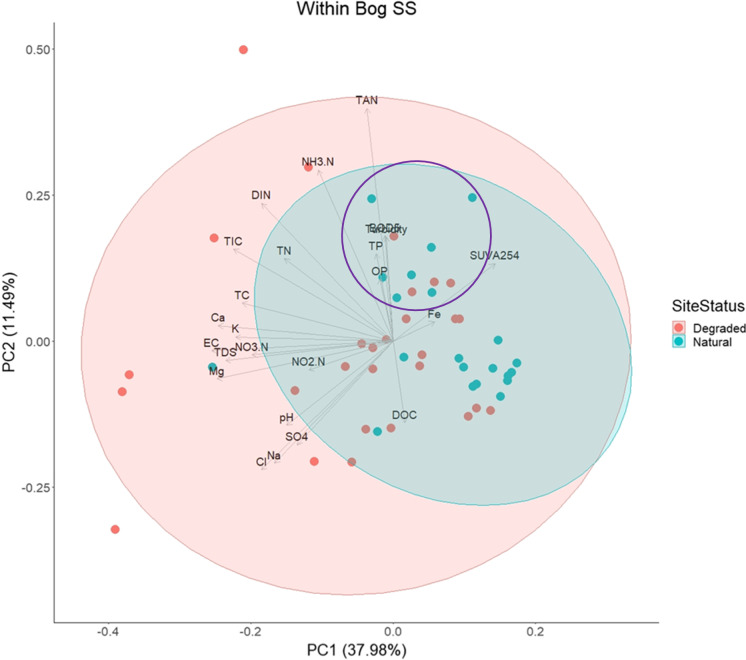
Biplot of principal component analysis (PCA) for *within bog small stream (SS)* data comparing degraded (red) and near-natural (blue) sites. Sites within the purple ellipse are associated with natural bogs that have been partially restored the longest (> 15 years) (see Online Resource 1)

Statistically significant (*P* ≤ 0.05) positive relationships (*r* ≥ 0.7) were associated with Cl, Mg, TC and TIC for a range of other water quality variables (Fig. 3). As well as being correlated with TC and TIC, Ca was also strongly correlated with EC and TDS. EC and TDS were also strongly correlated with each other. Finally, strong correlations exist between some nitrogen variables (DIN with TN and NO_3_–N, and TAN and NH_3_–N). The analysis for multicollinearity led to the exclusion of the following variables from further ANOVA analysis: Cl, Ca, TIC, Mg and TDS which are all correlated and clustered around EC (Fig. 4) while DIN and NO_3_–N and were removed being correlated with TN (Fig. 3).

### Seasonal variability in water quality

Following rigorous quality control of the sampling protocol, and chemical and statistical analyses, a final database of 156 samples were examined for 25 chemical elements. Meteorological conditions were considered normal vis-à-vis the 30-year average in terms of mean air temperature, whereas autumn 2020 was 7.3% wetter and autumn 2021 was 7% drier than the 30-year average (Mullingar Met Éireann station, met.ie). In addition, the location of all sampling sites located more than 40 km away from the coast ensures that marine influence was insignificant (Beltman et al., 1993).

While sampling periods were limited (two for autumn and one for spring), there was evidence of some seasonal variation in water quality (Table 2), with *RS* being more variable between seasons than *within bog SS*. In spring, *RS* water pH (*P* ≤ 0.001), EC (*P* ≤ 0.001) and TDN (*P* ≤ 0.001) were significantly greater when compared to autumn. In contrast, *RS* as well as *within bog SS* waters sampled in autumn had significantly greater concentrations of Fe (*P* ≤ 0.001 and 0.016 respectively). The SUVA_254_ value was also significantly lower in spring for both water types (*RS* *P* = 0.048; *within bog SS* *P* ≤ 0.001), which suggests that the DOC that reaches waters is more labile in spring than in autumn.

**Table 2:** Mean (± standard error) values of water quality parameters comparing seasons: spring (*n* = 1) and autumn (*n* = 2). Bold values signify significant differences (*P* ≤ 0.05) with 95% confidence (ANOVA 1). Variables excluded from the analysis due to multicollinearity are in italics

Parameter	*Within Bog SS*		*RS*	
	Spring	Autumn	Spring	Autumn
pH	7.26 ± 0.50	6.90 ± 0.51	**7.71 ± 0.07**	**7.31 ± 0.07**
EC (μS/cm)	281.58 ± 38.77	250.57 ± 35.54	**502.94 ± 25.73**	**407.30 ± 27.85**
TDS (ppm)	*126.96* ± *18.97*	*132.64* ± *20.64*	*238.91* ± *14.54*	*196.85* ± *14.20*
Turbidity (NTU)	5.51 ± 1.01	3.12 ± 0.70	3.91 ± 0.56	5.48 ± 0.94
TIC (mg/l)	*25.00* ± *3.92*	*19.01* ± *3.31*	*61.93* ± *5.22*	*40.81* ± *2.79*
DOC (mg/l)	31.44 ± 2.54	31.61 ± 1.88	23.82 ± 1.60	25.06 ± 1.35
SUVA_254_ (L/mg/m)	**3.17 ± 0.24**	**4.37 ± 0.17**	**2.34 ± 0.22**	**3.33 ± 0.23**
TDN (mg N/L)	1.59 ± 0.20	1.65 ± 0.14	**2.81 ± 0.22**	**1.77 ± 0.16**
TAN (mg N/L)	0.44 ± 0.10	0.44 ± 0.13	0.11 ± 0.03	0.10 ± 0.03
NH_3_–N (mg N/L)	*0.001* ± *0.000*	*0.001* ± *0.000*	*0.001* ± *0.000*	*0.000* ± *0.000*
NO_3_–N (mg N/L)	*0.70* ± *0.17*	*0.63* ± *0.14*	*2.10* ± *0.24*	*1.05* ± *0.17*
NO_2_–N (mg N/L)	0.01 ± 0.00	0.02 ± 0.01	0.01 ± 0.00	0.01 ± 0.00
DIN (mg/l)	*1.12* ± *0.18*	*1.17* ± *0.22*	*2.22* ± *0.24*	*1.15* ± *0.17*
TDP (mg/l)	0.01 ± 0.01	0.01 ± 0.01	0.00 ± 0.00	0.01 ± 0.00
OP (mg/l)	0.01 ± 0.01	0.00 ± 0.00	0.01 ± 0.00	0.01 ± 0.00
Cl (mg/l)	*13.29* ± *1.77*	*10.58* ± *0.87*	*6.85* ± *2.47*	*15.69* ± *0.89*
SO_4_ (mg/l)	8.59 ± 2.05	16.95 ± 4.09	12.00 ± 1.76	18.70 ± 2.78
Na (mg/l)	6.81 ± 0.67	6.87 ± 0.23	8.26 ± 0.42	8.52 ± 0.36
K (mg/l)	0.69 ± 0.13	0.56 ± 0.08	1.96 ± 0.23	2.40 ± 0.20
Mg (mg/l)	*2.58* ± *0.38*	*2.30* ± *0.30*	*4.84* ± *0.35*	*5.43* ± *0.41*
Ca (mg/l)	*55.95* ± *8.62*	*45.97* ± *7.98*	*96.78* ± *6.62*	*95.37* ± *6.59*
Fe (µg/l)	**280.73 ± 37.90**	**545.61 ± 85.72**	**245.02 ± 47.68**	**732.06 ± 172.49**
BOD (mg/l O_2_)	1.89 ± 0.32	2.50 ± 1.15	0.78 ± 0.27	0.68 ± 0.21

### Within bog small streams in degraded and natural bogs

The *within bog SS*_nat_ had a pH range of 5.8 − 7.9 units, with EC values oscillating between 30 and 520 μS/cm and TDS values between 18  and  82 ppm. The *within bog SS*_deg_ had pH values that ranged between 2.9 and  7.9 units, EC values that ranged between 50 and 730 μS/cm and TDS between 20 and 428 ppm.

Turbidity values ranged between 0.7 and 23 NTU in *within bog SS*_nat_ and between 0.6 and 18 NTU in *within bog* SS_deg_. DOC concentrations and SUVA_254_ values for *within bog SS*_nat_ ranged from between 12 and 64 mg/l and 2.10−5.19 l/mg/m, respectively, whereas DOC in *within bog* SS_deg_ ranged between 11 and 53 mg/l and SUVA_254_ ranged between 0.70 and 6.07 l/mg/m. TDN concentrations were 0.4 − 2.2 mg N/L for *within bog SS*_nat_ and 0.6 − 3.9 mg N/L for *within bog* SS_deg_, with TAN concentrations ranging from 0.01 to 1.91 and 0.02 − 2.88 mg N/L respectively.

Concentrations of other ions in *within bog SS*_nat_ ranged from 1 to 14 mg/l for SO_4_, 5−11 mg/l for Na, 0.2 − 2.9 mg/l for K, 0.8−5.9 mg/l for Mg, 6−138 mg/l for Ca and 114−1090 µg/l for Fe in comparison to SS_*deg*_ values which varied much more widely: 0.4 − 79 mg/l for SO_4_, 1 − 20 mg/l Na, 0.03 − 2.3 mg/l K, 0.8−9 mg/l Mg, 4−147 mg/l Ca and 25−2310 µg/l Fe.

A multivariate approach using PCA was used to visualise the variation in the data (*b* ≤ 0.001, KMO = 0.5). The first PCA explored *within bog SS* data by comparing water quality between the *within bog SS*_deg_ and *within bog SS*_nat_ (Fig. 4). Of the 25 PCs (principal components) produced, PC1 and PC2 explained 50% of the variance. The variables with greater loading values (> ± 0.30; Online Resource 7) and, therefore, contributing the most to PC1 were EC, Mg, Ca, and TDS, all with negative loadings, whilst SUVA_254_ had the greatest positive influence, although with a weaker loading value of 0.18. For PC2, N parameters (TAN, NH_3_–N and DIN) had the most positive influence, whilst Cl and Na had the greatest negative influence (loadings − 0.27 and − 0.26 respectively).

Overall, *within bog SS*_nat_ sites had a distinct cluster that was characterised by greater positive loading values for SUVA_254_ and negative loading values for all pollutants, EC and pH. In contrast, *within bog SS*_deg_ sites were less tightly clustered and were most strongly influenced by TAN, NH_3_–N and SO_4_ loadings (PC2), as well as by greater EC, Mg and Ca values (PC1). Of note, a sub-group of *within bog* SS_nat_ were directly associated with decreasing loading values for DOC and increasing TAN (Fig. 4). These sites (Moyclare, Raheenmore, Carrowganappul and Mount Hevey bogs) have been partially restored > 15 years (i.e. the longest) (Online Resource 1). Finally, one degraded site (Drinagh) was responsible for the Na and Cl loadings revealing a possible salt pollution event from the ‘gritting’ of nearby surfaces before our spring 2021 sampling (Na = 20 mg/l and Cl = 53 mg/l).

**Fig. 5:**
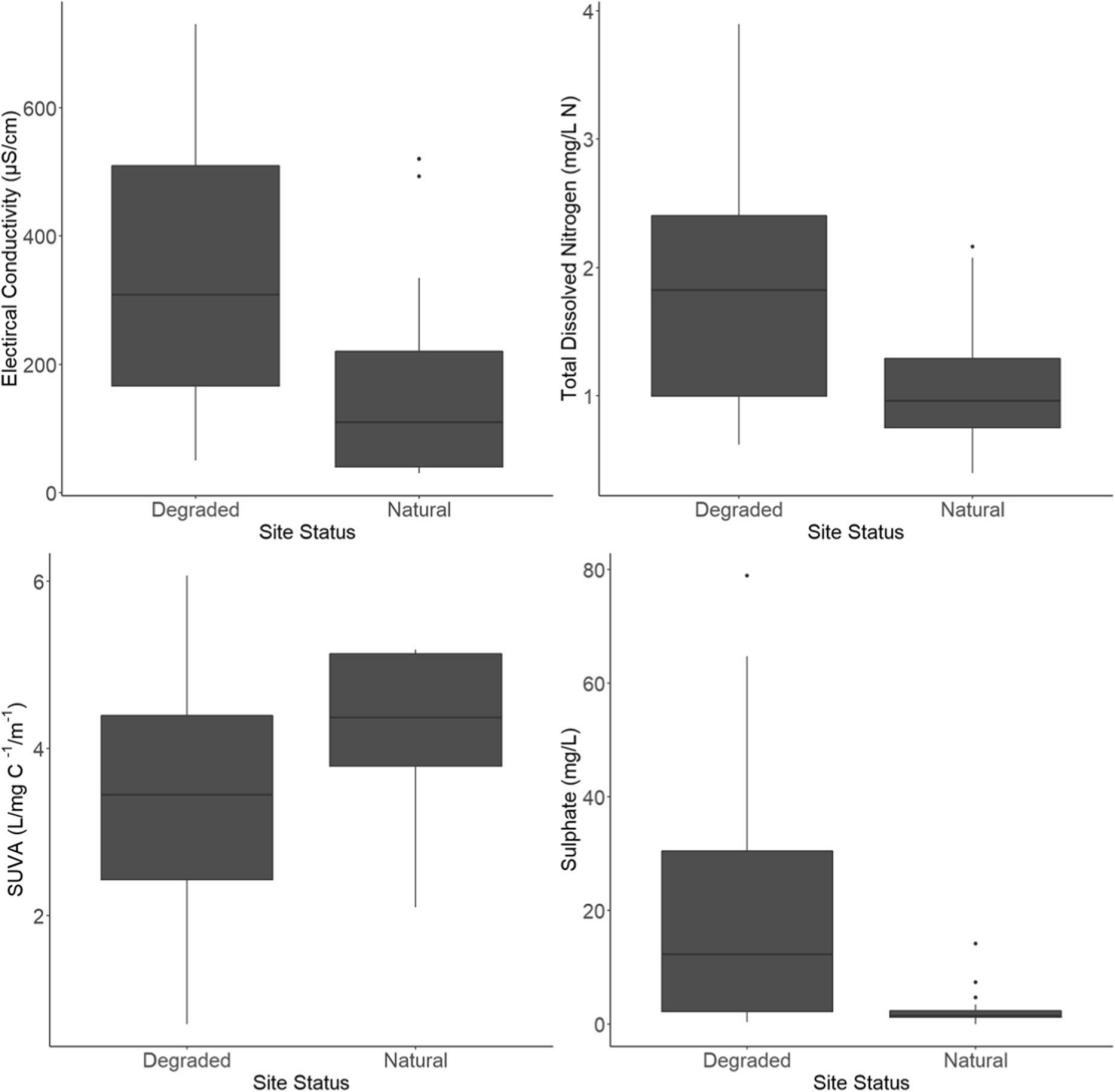
Boxplots comparing degraded (*within bog SS*_*deg*_) and natural (*within bog SS*_*nat*_) chemistry data for all sampling periods, where statistically significant differences were found (*P* ≤ 0.05) with 95% confidence (ANOVA 2, Online Resource 4)

Of the water quality parameters analysed, the *within bog SS*_*deg*_ (Table 3, *N* = 28) had significantly greater mean concentrations/values of EC (*P* ≤ 0.001), TDN (*P* = 0.048), and SO_4_ (*P* ≤ 0.001) compared to *within bog SS*_nat_ (Fig. 5; Table 3; Online Resource 4). The mean SUVA_254_ value was significantly lower for *within bog SS*_deg_ (3.33 ± 0.24 l/mg/m) compared to *within bog SS*_nat_ (4.34 ± 0.18 l/mg/m) (*P* ≤ 0.001). Similarly, as described above, PCA analysis also found EC and SUVA_254_ to be influential variables. DOC and TDS, alongside other major ions (TAN, NO_3_) and base cations (Na, K, Ca, Cl, Mg) had all greater concentrations for *within bog SS*_deg_ but these were not statistically significant, or they were not tested due to multicollinearity within the ANOVA model. Fe concentrations and BOD were greater in *within bog SS*_nat_ but these were found not to be significant.

**Table 3:** Mean (± standard error) values of water quality parameters for degraded (*within bog SS*_deg_, *n* = 17) and natural (*within bog SS*_nat_, *n* = 11) bogs. Bold values signify significant differences (*P* ≤ 0.05) with 95% confidence (ANOVA 2, Online Resource 4). Variables excluded from the analysis due to multicollinearity are in italics

Parameter	Degraded	Natural
pH	7.04 ± 0.14	6.96 ± 0.11
EC (μS/cm)	**334.40 ± 31.57**	**155.64 ± 27.03**
TDS (ppm)	*170.76* ± *16.98*	*64.44* ± *11.12*
Turbidity (NTU)	4.11 ± 0.71	4.61 ± 1.22
TIC (mg/l)	*24.02* ± *3.08*	*14.89* ± *3.27*
DOC (mg/l)	34.74 ± 1.65	27.14 ± 2.20
SUVA_254_ (l/mg/m)	**3.33 ± 0.24**	**4.34 ± 0.18**
TDN (mg N/L)	**1.89 ± 0.15**	**1.04 ± 0.09**
TAN (mg N/L)	0.48 ± 0.10	0.35 ± 0.12
NH_3_–N (mg N/L)	*0.001* ± *0.00*	*0.000* ± *0.00*
NO_3_–N (mg/l)	*0.76* ± *0.17*	*0.53* ± *0.13*
NO_2_–N (mg/l)	0.02 ± 0.01	0.01 ± 0.00
DIN (mg/l)	*1.29* ± *0.20*	*0.89* ± *0.17*
TDP (mg/l)	0.01 ± 0.01	0.02 ± 0.01
OP (mg/l)	0.00 ± 0.00	0.01 ± 0.01
Cl (mg/l)	*13.49* ± *1.59*	*9.84* ± *0.82*
SO_4_ (mg/l)	**18.49 ± 3.31**	**2.58 ± 0.67**
Na (mg/l)	7.19 ± 0.57	6.25 ± 0.35
K (mg/l)	0.71 ± 0.10	0.55 ± 0.13
Mg (mg/l)	*2.84* ± *0.35*	*1.83* ± *2.56*
Ca (mg/l)	*61.80* ± *7.90*	*34.39* ± *7.02*
Fe (µg/L)	351.14 ± 68.78	543.48 ± 60.37
BOD (mg/l O_2_)	1.77 ± 0.30	3.05 ± 1.51

When assessing the interactions between site condition (natural vs degraded) and sampling periods (ANOVA 2), individual sampling periods were not found to influence the significant differences found between degraded and natural sites (Online Resource 4).

### Changes in water quality in receiving streams

A total of 34 sites were sampled on each occasion from receiving streams (*RS*), where sampling took place upstream and/or downstream of a *within bog SS* tributary (Fig. 2).

**Fig. 6:**
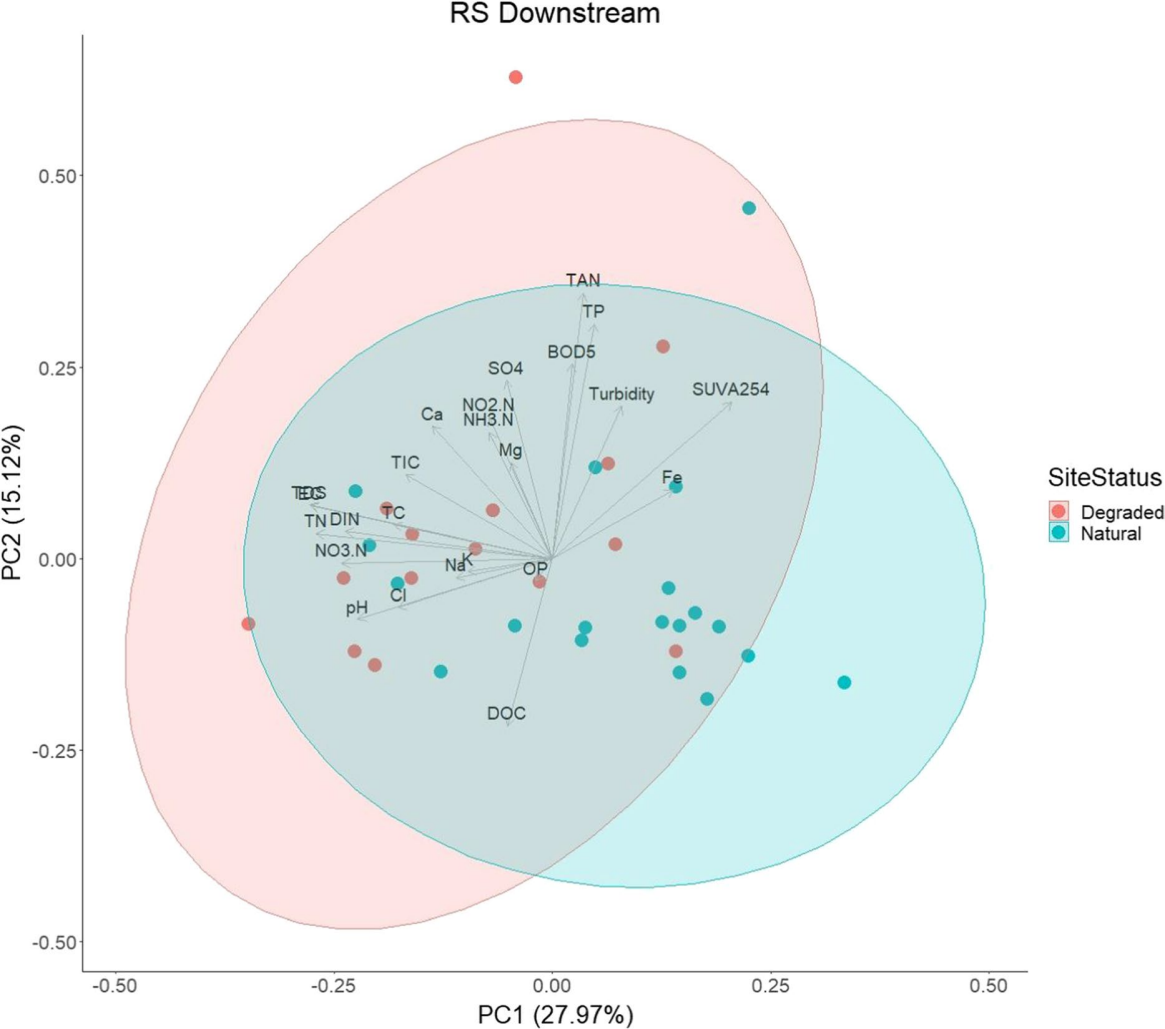
Biplot of principal component analysis (PCA) for receiving stream (*RS*) downstream of degraded (red) and natural (blue) bog sites

A PCA (PCA2) was performed to explore water quality data for streams that receive water downstream of the bog (both degraded and natural sites) tributaries (*RS downstream*) (*b* ≤ 0.001, KMO = 0.5) (Fig. 6). Of the 24 PCs produced, PC1 and PC2 explained 43% of the variance. For PC1, TDS, EC, TDN, NO_3_–N and DIN contributed the most (with loading values > -0.30; Online Resource 6), whilst variables TAN, TDP and BOD contributed the most for PC2 (with loading values > 0.30). There was little distinction in the clustering of degraded and natural sites, which suggests limited variation between them.

With the exception of pH, Mg, and variables with negligible concentrations (NH_3_–N, NO_2_–N, and OP), mean concentrations of all other parameters increased downstream of degraded sites (Table 4) corresponding with the findings of the PCA analysis (parameters with high loading values). Some parameters also increased downstream of natural sites (EC, TDS, TIC, TDN, NO_3_–N, Na, K, Mg, Fe). However, based on the results of ANOVA tests on bulked data (ANOVA 3a and b, Online Resources 5 and 6), there were no significant differences in water quality parameters between upstream and downstream of either degraded or natural sites. Of note, DOC concentrations across all receiving streams ranged from 7.2 to 63.9 mg/l with a mean value of 27.91 mg/l and a median value of 27.26 mg/l, with lowest and highest values being recorded from the same downstream river of a natural bog (Garriskill). Unionised ammonia concentrations were negligible (with a maximum value of 0.008 mg N/L across all data) at the time of analysis. However, variables such as temperature, pH and Ca concentrations can impact NH_3_–N production and concentration, and concentrations may fluctuate during different seasons and hydrological conditions.

**Table 4:** Mean and ± Standard Error values of water quality parameters for receiving streams (*RS*) upstream and downstream of degraded and natural bog sites. No significant differences were found for either degraded or natural sites (ANOVA 3a and b, Online Resources 5 and 6). Variables excluded from the analysis due to multicollinearity are in italics

Parameter	Degraded		Natural	
	Upstream	Downstream	Upstream	Downstream
pH	7.55 ± 0.10	7.54 ± 0.11	7.43 ± 0.09	7.40 ± 0.14
EC (μS/cm)	484.15 ± 43.97	524.86 ± 31.87	353.56 ± 38.89	415.10 ± 26.88
TDS (ppm)	*226.5* ± *24.98*	*256.24*	*171* ± *19.52*	*201.10* ± *13.50*
Turbidity (NTU)	2.91 ± 0.47	4.65 ± 1.07	5.96 ± 2.23	5.95 ± 1.07
TIC (mg/l)	*48.65* ± *5.40*	*58.33* ± *7.13*	*39.38* ± *4.19*	*46.65* ± *3.50*
DOC (mg/l)	23.92 ± 1.77	24.59 ± 1.77	25.15 ± 2.08	26.35 ± 2.13
SUVA_254_ (l/mg/m)	2.32 ± 0.35	2.65 ± 0.35	3.92 ± 0.33	3.12 ± 0.33
TDN (mg N/L)	2.19 ± 0.34	2.54 ± 0.26	1.75 ± 0.20	1.90 ± 0.19
TAN (mg N/L)	0.10 ± 0.03	0.17 ± 0.06	0.08 ± 0.02	0.09 ± 0.02
NH_3_–N (mg N/L)	*0.001* ± *0.000*	*0.001* ± *0.000*	*0.000* ± *0.000*	*0.001* ± *0.000*
NO_3_–N (mg/l)	*1.56* ± *0.43*	*1.73* ± *0.33*	*1.28* ± *0.29*	*1.48* ± *0.22*
NO_2_–N (mg/l)	0.01 ± 0.00	0.02 ± 0.01	0.01 ± 0.00	0.01 ± 0.00
DIN (mg/l)	*1.59* ± *0.40*	*2.00* ± *0.34*	*1.36* ± *0.28*	*1.58* ± *0.22*
TDP (mg/l)	0.00 ± 0.00	0.01 ± 0.00	0.02 ± 0.01	0.01 ± 0.00
OP (mg/l)	0.01 ± 0.01	0.00 ± 0.00	0.02 ± 0.01	0.00 ± 0.00
Cl (mg/l)	*18.57* ± *1.60*	*20.27* ± *2.03*	*14.77* ± *0.98*	*15.02* ± *0.84*
SO_4_ (mg/l)	16.20 ± 1.44	26.72 ± 5.19	8.94 ± 1.61	8.85 ± 1.07
Na (mg/l)	9.22 ± 0.55	9.66 ± 0.77	7.37 ± 0.31	7.71 ± 0.24
K (mg/l)	2.16 ± 0.26	2.27 ± 0.22	1.82 ± 0.46	2.27 ± 0.28
Mg (mg/l)	*6.30* ± *0.54*	*5.69* ± *0.37*	*3.55* ± *0.46*	*4.73* ± *0.45*
Ca (mg/l)	*99.99* ± *11.10*	*113.77* ± *6.55*	*85.16* ± *10.52*	*89.30* ± *7.46*
Fe (µg/l)	222.03 ± 41.77	500.63 ± 262.35	663.57 ± 182.25	953.66 ± 283.37
BOD (mg/l O_2_)	0.79 ± 0.32	1.1 ± 0.56	0.92 ± 0.40	0.65 ± 0.25

### Comparing upstream and downstream *RS* quality in individual sites

**Fig. 7:**
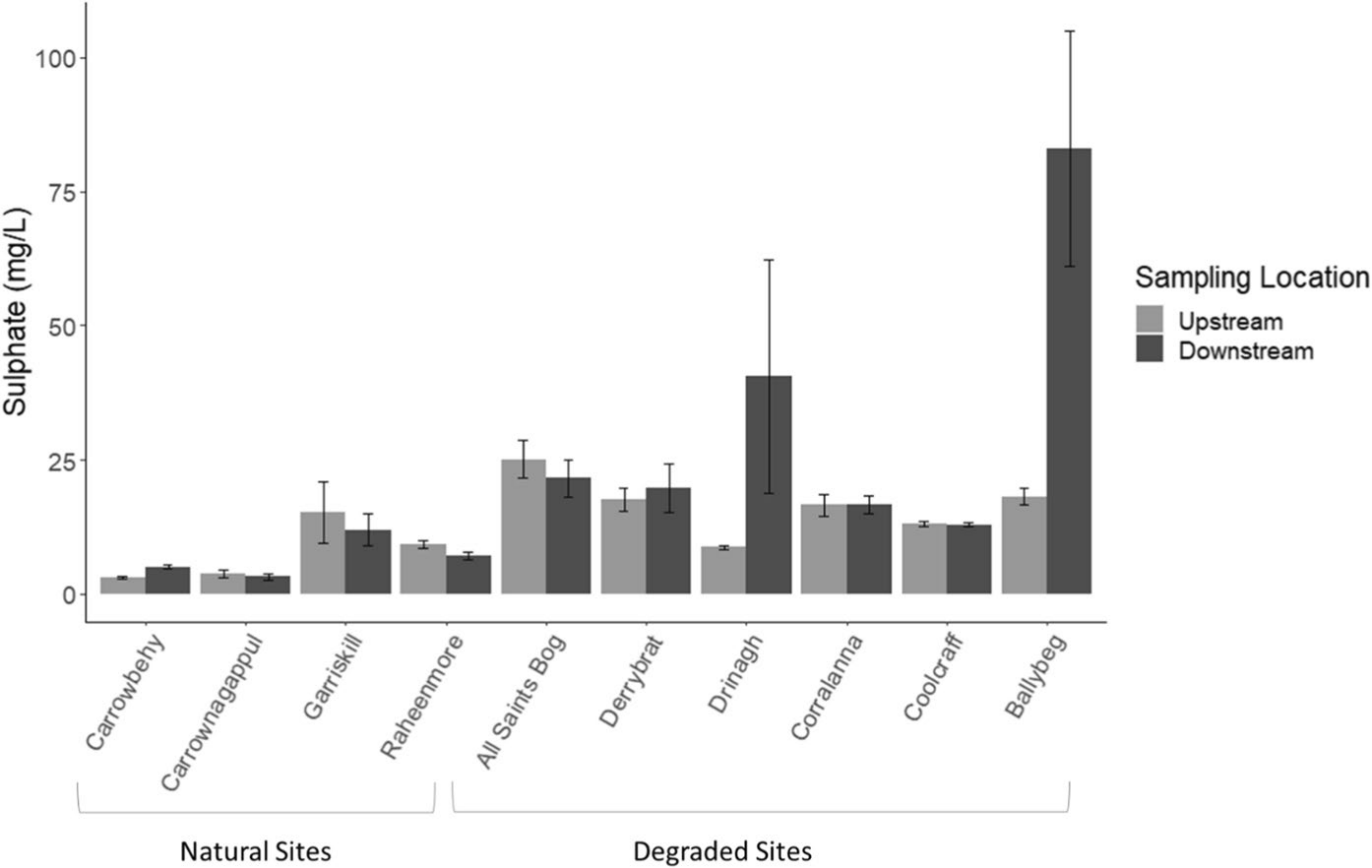
Bulked mean concentration of sulphate (SO_4_) upstream and downstream of individual bogs

**Fig. 8:**
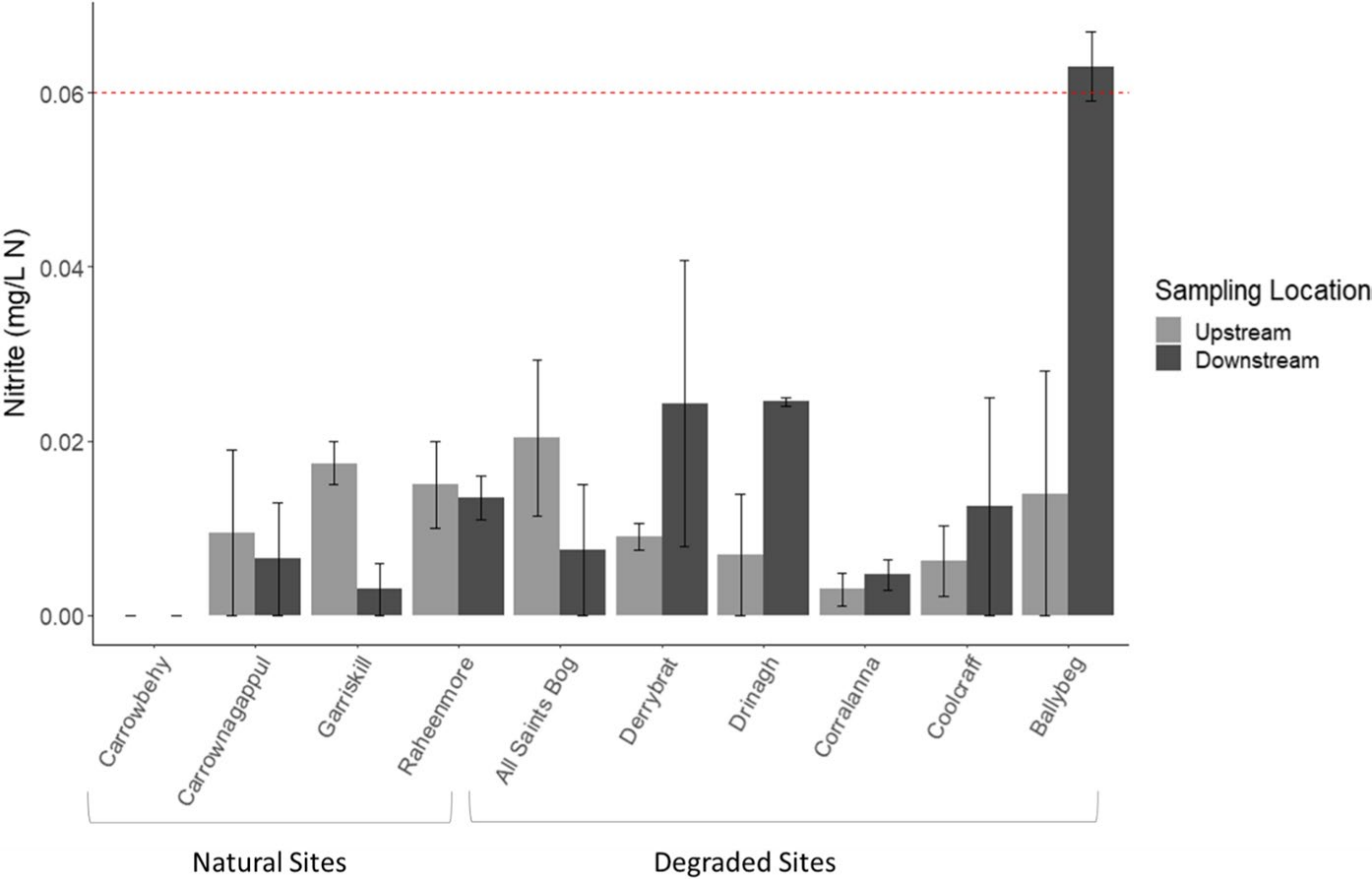
Bulked mean concentration of nitrite (NO_2_–N) upstream and downstream of individual bogs. Red dashed line represents EPA guide value (0.06 mg N/L) for Ireland

**Fig. 9:**
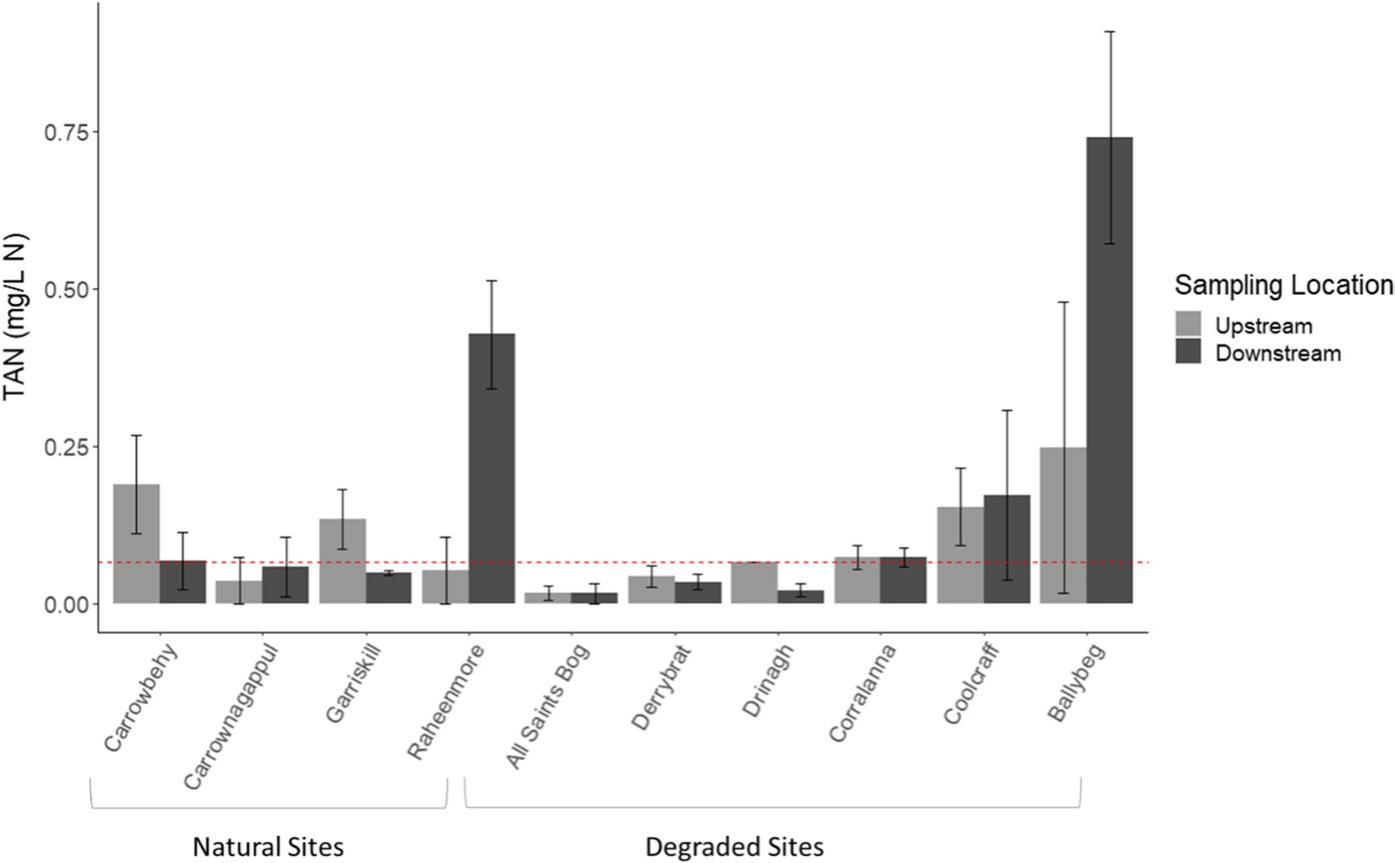
Bulked mean concentration of total ammonia (TAN) upstream and downstream of individual bogs. Red dashed line represents the threshold for good ecological status (0.065 mg N/L) based on EPA Environmental Quality Status (EQS) for Ireland

**Fig. 10:**
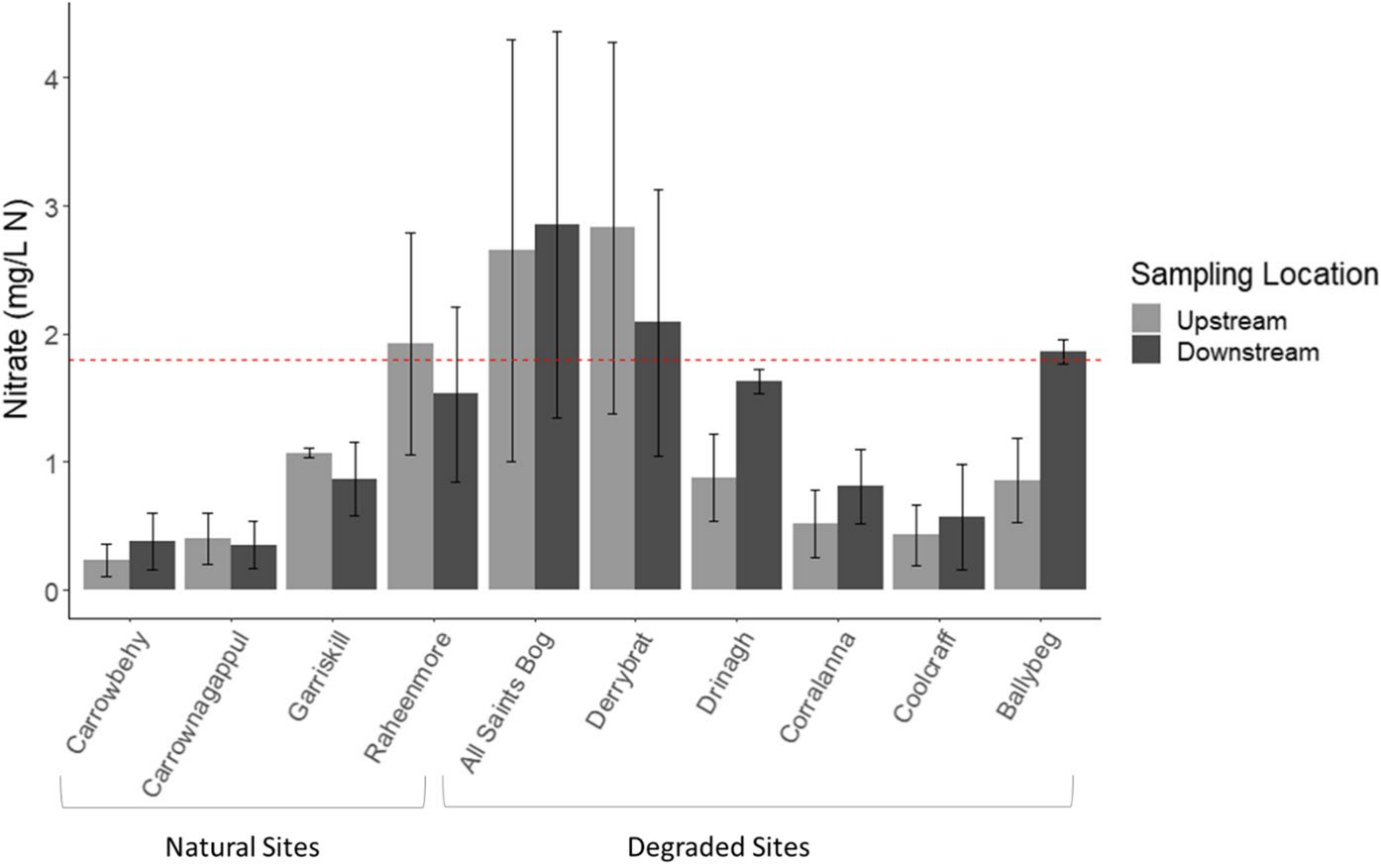
Bulked mean concentration of nitrate (NO_3_–N) upstream and downstream of individual bogs. Red dashed line represents the threshold for good ecological status (1.8 mg N/L) based on EPA Environmental Quality Status (EQS) for Ireland

Figures 7, 8, 9 and 10 present the variables where there were high concentrations upstream or downstream of individual bogs, or of elements which are legislated for. Greater concentrations of SO_4_ (*P* ≤ 0.001; 83.04 ± 21.92 mg/l; Fig. 7) were measured downstream of Ballybeg, a degraded site, when compared to upstream (18.16 ± 1.55 mg/l), which suggests that the water chemistry of the Coolcar Stream within the Yellow River Catchment (north-east midlands) is impacted by this drained bog. Water samples taken downstream of the degraded Drinagh bog also had greater SO_4_ concentrations (*P* ≤ 0.001; 40.50 ± 21.66 mg/l) when compared to upstream (8.67 ± 0.37 mg/l).

Downstream of Ballybeg, concentrations of both NO_2_–N (0.06 ± 0.00 mg N/L) and TAN (0.74 ± 0.17 mg N/L) were above the “good status” Environmental Quality Status (EQS) for Ireland (EQS values being 0.06 and 0.065 mg N/L respectively (EPA, 2011a); Figs. 8 and 9). TAN concentrations were above the guideline thresholds at two other degraded sites (both upstream and downstream), also located in the north-east midlands within the Inny River catchment (Corralanna and Coolcraff). Of note, TAN concentrations downstream of Raheenmore (a natural bog) were greater than the high-status guideline concentration by one order of magnitude (Fig. 9) with a downstream mean value 0.43 ± 0.09 mg/l, compared to an upstream mean of 0.05 ± 0.05 mg/l.

Greater NO_3_–N concentrations were recorded for waters sampled from *RS* compared to *within bog SS*, with mean concentrations from the degraded sites just below the threshold for good status (1.8 mg N/L, (EPA, 2011a)). When comparing upstream and downstream *RS* for individual sites, Ballybeg, All Saints Bog and Derrybrat exhibited NO_3_–N concentrations greater than the 1.8 mg N/L threshold for both upstream and downstream waters (Fig. 10), although there was large standard error in the data associated with temporal variation.

Finally, while the overall BOD means remained below the “high status” EQS (< 1.3 mg O_2_/L; SI 272 2009), values were consistently exceeded in downstream individual samples from two degraded sites (Ballybeg and Corralanna) where BOD reached a mean of 5 and 6 mg O_2_/L respectively over the monitoring period (data not shown).

## Discussion

This study provides, for the first time, an in-depth analysis of the large array of chemical parameters found in streams within a degraded (drained mostly from peat extraction) bog landscape, thereby yielding robust evidence of the relatively poor water quality status of small streams and rivers that flow through the Irish midlands. This region was once home to the largest area of oceanic raised bog habitat, but recent national monitoring surveys have shown that of the original 310,000 ha found in this region, 260,000 ha (or 84%) have been affected by peat extraction (both industrial extraction and/or domestic turf cutting) (NPWS, 2017). This study supports an increasing body of literature supporting the fact that sustainable management of peatlands should be given high priority in countries where degraded and/or drained peatlands are a key land use category.

### Overall water quality of streams within a degraded bog landscape

Although raised bogs are an acidic soil type (Renou-Wilson et al., 2022), the pH of all the sampled streams in our study can be qualified as circum-neutral or alkaline (*within bog SS* pH > 6 and *RS* pH > 7) and did not differ across bog status and stream sampling locations. This reflects a small influence from natural bog water in contact with acidic and anaerobic peat layers in favour of water in contact with the sub-peat substrate of lacustrine origin or the limestone geological parent material found in this region (Feehan et al., 2008). In our study, the median value for *within bog SS*_nat_ was 110 µS/cm, a typical value for raised bogs found in other site-specific Irish studies (Schouten, 2002). However, EC levels were significantly greater in *within bog SS*_deg_ with a median value that was four times greater (470 µS/cm) and this was also visible in the elevated concentrations in *downstream RS*. Drainage has allowed bog water to come into contact with the high pH, sub-peat, mineral material (till and limestone) with which it can react. It is also likely that older drained and extracted bogs are now under the influence of regional ground water, which can discharge into the bog stream at its periphery. This is supported by the significant positive correlation observed here between EC and Ca.

The chemical composition of the streams sampled in this study reflects the decreased influence from typical natural bog water (in contact with nutrient-poor, acidic peat layers) and thus confirms the widespread degradation of these ecosystems in this region. More critically, higher pH, EC and Ca values would indicate waters associated with fen peat rather than ombrotrophic peat, which has serious implications for the rewetting and restoration of these degraded raised bog ecosystems. A neutral pH is an optimum environment for peat mineralisation, especially where nutrients are available. Therefore, it is crucial that drainage-induced degradation ceases as soon as possible, and that bogs are rewetted at a minimum and restored at best so as to recover their biogeochemical functioning (Emsens et al., 2020; Urbanová & Bárta, 2020) and associated water chemistry.

### Nitrogen and peatland management

In this study, total DIN was fully taken into account as we quantified TAN, NO_3_–N and NO_2_–N. Here, DIN was predominantly dominated by NO_3_, except in the smaller *within bog SS* sites where a considerable amount of TAN was also present (NO_2_–N was not an important species at any site). This profile is comparable to other studies from peatlands in the UK (Adamson et al., 1998) and Canada (Andersen et al., 2010).

However, it is important here to differentiate between *within bog SS* and *downstream RS,* not just in terms of chemical species distribution but also in terms of absolute values. Across all sampling locations, TAN concentrations ranged from 0.014 to 2.88 mg N/L, with only 57% of the sampling locations exhibiting a better than *good* water quality EQS (0.065 mg N/L, SI 272 2009). Mean TAN concentrations were greatest in *within bog SS* where they were 7 and 5 times the EQS for degraded and natural bogs, respectively. These levels are greater than those typically recorded in bog waters from northern and central Europe but similar levels have been found in less natural bogs in Canada and Northern United States (Bourbonniere, 2009; Andersen et al., 2010). Meanwhile the receiving streams in our study contained greater TAN concentrations (0.17 mg N/L) downstream from degraded bogs compared to natural bogs (0.09 mg N/L), although not statistically significant. Receiving streams from degraded sites typically varied but very high concentrations were recorded at specific sites such as Coolcraff and Ballybeg. Concentrations of unionised ammonia (NH_3_–N) were low (< 0.01 mg N/L) at all sites. However, the fraction of NH_3_–N is known to increase with increasing pH, which is problematic as NH_3_–N is toxic to aquatic organisms (Alonso & Camargo, 2015). While there is no EQS value for NH_3_–N, concentrations of 0.02 − 0.10 mg N/L are considered safe for aquatic organisms (Hickey et al., 1999; Levit, 2010; Zhang et al., 2018). While the concentrations measured during the three sampling periods in this study fell below thresholds to warrant concern for aquatic organisms, this may change during the summer period, as temperature increases the NH_3_–N fraction. In addition, an uptrend in the pH in receiving streams due to additional pollution pressures and additional alkaline groundwater input from limestone geology would also be problematic and should be closely monitored.

Across the studied region, mean upstream NO_3_–N concentrations were twice that of their respective *within bog SS,* which remained relatively low. Greater mean NO_3_–N concentrations were recorded downstream of both degraded and natural sites with highest means found in *RS downstream* from the degraded sites, reaching levels just below the “good status” EQS. Apparent downstream increase in both NO_3_–N and TAN concentrations in *RS* of degraded sites seems related to the respective *within bog SS* proportional contributions to the receiving stream environments. Greater levels of TAN contaminant in the degraded sites *RS* suggest potential harmful consequences further downstream due to additional nitrification. It is not clear whether all the TAN detected in the *within bog SS* is nitrified or due to differential uptake rates between receiving streams, amongst other factors.

While mineralisation and nitrification processes occur in both degraded and natural bogs, greater (statistically significant) TDN concentrations were found in *within bog SS*_deg_ (1.89 mg N/L) compared to *within bog SS*_nat_ (1.04 mg N/L). Since the relative contribution of DON (calculated as TDN-DIN) was greater than mean TDN at degraded compared to natural within bog SS, this could suggest additional leaching of organic N. This dissolved organic N is in the form of macromolecules, and result from the accelerated breakdown of larger organic N compounds into smaller units. This is likely due to rapid, increased aerobic conditions during water table fluctuations in drained bogs (Laine et al., 2013).

Raised bogs are ombrotrophic mires with a water composition typically similar to local rainfall (Proctor, 1992). The most recent rainfall analyses for the Irish midlands report TAN concentrations of < 0.4 mg N/L and DOC of < 2 mg/l (Aherne & Farrell, 2002; Aherne, 2016). Both *within bog SS* and *RS* within this bog landscape are N enriched. This snapshot picture implies that even the ‘best conservation status’ raised bog SACs in Ireland are leaching relatively high levels of N. The water chemistry of the *within bog SS*, in particular, has direct implications for the successful restoration of these sites. Firstly, these designated natural bogs continue to experience the effects of long-term drainage, which has led to organic matter decomposition and mineralisation of nutrients, in this case the carbon-bound N. While it was expected that decomposition by microbial activity would be impeded in the natural bogs of this study (due to rewetting from dam blocking as part of the restoration plans), the water table may still be too low or unstable, especially in the summertime. This has been experienced in other rewetted bogs in this region (Renou-Wilson et al., 2018, 2019).

Secondly, N saturation occurs when the sum of TAN, NO_3_ and NO_2_ is in excess of the nutritional requirements by the plant and microbial biomass (Aber et al., 1989). In natural bogs, this would occur when the supply of nitrogenous compounds from the atmosphere exceeds the demand for these by the flora and soil fauna. While the analysis of precipitation was not carried out for the sampled sites at the time of measurement, recent atmospheric ammonia measurements at two sites (Garriskill and Raheenmore) show low (1.18 μg NH_3_/m^3^) and high (2.34 μg NH_3_/m^3^) concentrations, respectively (Kelleghan et al., 2021). Ammonia has been linked to microbiological shifts on bogs from typical bacteria and fungi to the proliferation of algae (Payne et al., 2013). The Raheenmore site has seen an increase of algae slime growing on heather and while nitrogen sensitive species such as *Ramalina* spp. were still present, they are also being encroached on by algae (Kelleghan et al., 2022). This is corroborated by the elevated TAN concentrations found downstream from the Raheenmore SAC (0.43 mg N/L). This warrants a close monitoring of TAN in SAC bog streams in conjunction with local aerial N deposition.

### Fluvial carbon dynamics and implications

Greater (28%) DOC concentrations were observed in *within bog SS*_*deg*_ compared to natural/restored bog sites albeit not statistically significant. The range of values for natural sites (11 − 63 mg/l) are typical for the region (Regan et al., 2020) as well as for international similar sites (Moore & Clarkson, 2007; Roulet et al., 2007; Gaffney et al., 2020). The greater values are more reflective of rewetted (previously extracted) sites (Evans et al., 2016c). Of interest, however, was that DOC concentrations in all the receiving streams were high (overall median value 27.2 mg/l) compared to other Irish streams, even within other peatland catchments: Liu et al (2014) report DOC values (median of 5.7 mg/l) from 55 streams across a 12-month period with the highest mean values recorded from watersheds dominated by peat soils (14.5 mg/l), while DOC from streams at a natural blanket bog in the west of Ireland averaged 11.5 mg/l (Koehler et al., 2009). DOC values in our study region have been shown to be even greater than other regions with drained peatlands. For example, mean concentrations from both forested and non-forested peatland catchments in the west of Ireland were all < 23 mg/l (Ryder et al., 2014; Kelly-Quinn et al., 2016). The high upstream DOC concentrations in our study, regardless of the bog status of the tributary, can explain the lack of impact of within-bog water in this landscape. More critically, our results point towards a widespread loss of fluvial carbon within this region, resulting from (1) their location within a bog landscape (Fig. 1), and (2) the results of decades of peat drainage and extraction in industrial or domestic extracted peatlands in this region (since the 1950s). Such outcomes have also been reported in similar drained peatland catchments in other countries (Wallage et al., 2006; Waddington et al., 2008; Armstrong et al., 2010; Haapalehto et al., 2014; Evans et al., 2016c). Increasing DOC concentrations is a widespread phenomenon which has been associated with land use change and therefore managing the land use has been identified as the most practicable measures to counteract browning of natural waters (Kritzberg et al., 2020). However this may be hampered by other drivers contributing to such environmental change inter alia climate change (Wit et al., 2016).

Greater SUVA_254_ values are typically correlated with the aromatic and hydrophobic fractions, and the molecular weight of dissolved organic matter (DOM) (Weishaar et al., 2003; Spencer et al., 2012). The significantly greater SUVA_254_ value observed in *within bog SS*_nat_ indicates enhanced content of aromatic material within the DOC pool. This humic material has greater photo-reactivity, which leads to mineralisation of DOC to CO_2_ early in the headwater streams, as found in other peatland catchments (Franke et al., 2012; Pickard et al., 2017; Panneer Selvam et al., 2019). Higher temperatures may also be the cause of this DOC composition with increased temperature shown to be the leading factor explaining DOC variability in natural bogs (Rosset et al., 2022).

These carbon dynamics also have implications for regional drinking water quality. Natural bog streams with higher SUVA_254_ values also contain hydrophobic compounds which, together with lower alkalinity levels, are more easily removed by conventional surface water treatment technologies, such as coagulation processes. Hydrophilic compounds that have lower SUVA_254_ and higher alkalinity values are more difficult to treat, and pH regulation by acid addition may be necessary to achieve the required trihalomethane formation potential and chlorine demand reductions (EPA, 2011b; Casey et al., 2012). The degraded bog streams in our study also showed greater alkalinity levels, which combined with low SUVA_254_ values, would be less amenable to coagulation and more prone to biofouling of the filtration membranes (Archer & Singer, 2006). In addition, reprocessed/ microbial-delivered humic natural organic matter has been identified as the dominant component that remains after conventional treatment technologies both in Ireland (O'Driscoll et al., 2018) and internationally (Baghoth et al., 2011; Shutova et al., 2014), with implications for the final concentrations of trihalomethane in drinking water.

### Other chemical elements of interest

Enhanced peat decomposition is likely responsible for the significantly greater SO_4_ values observed in *within bog SS*_deg_ compared to *within bog SS*_nat_ (18.49 and 2.58 mg/l, respectively), with twice as much SO_4_ measured downstream of the degraded bogs compared to upstream, with one site (Ballybeg) recording four times the SO_4_ concentration. While SO_4_ deposition has been recorded on bogs elsewhere in the world from atmospheric origin (Andersen et al., 2010) and is leached out during drier periods, this is not the case for the Irish midlands where precipitation concentrations remain low (EBAS, 2022). However, adjacent agricultural enterprises may be responsible for some SO_4_ deposition, especially on the natural bog sites. Such monitoring would be needed to help with the appropriate assessment of activities within the vicinity of these protected SAC bogs. More critically, high SO_4_ concentrations have been shown to stimulate the production of methylmercury (MeHg) by sulphate-reducing bacteria, which could have serious implications for water quality and food chains downstream of these bogs (Branfireun et al., 1999; Bourbonniere, 2009). Another major anion, Cl, was unusually high at all sites compared to natural bog water elsewhere (Bourbonniere, 2009) and reflects the marine influence on the peat-forming vegetation across the island. Consequently, higher Cl content in within bog SS from degraded bogs may be attributed not only to high baseline level but also potential additional groundwater pollution from surrounding land use, as seen also in Canada (Andersen et al., 2011).

Higher concentrations of Mg and Ca in degraded bogs confirm the influence of nutrient-rich (higher pH) fen peat found at the base of the degraded bogs (Renou-Wilson et al., 2022), which is exposed after decades of peat extraction as well as being influenced now by groundwater which has a calcium-bicarbonate signature from a limestone geological influence (Feehan et al., 2008).

Low levels of phosphorus (P) were expected in all waters associated with raised bog peats (Renou-Wilson et al., 2022). This was confirmed for both TDP and orthophosphate (OP). While mineralisation of organic matter is expected to release small amounts of P in drained extraction bogs, it is likely bound to Fe, which exhibited elevated concentrations in all streams. Despite the potential desorption of P further downstream, the amount is likely to remain very low and is not of concern in this landscape.

Biological oxygen demand (BOD), an indicator of organic enrichment in the water, exhibited large temporal and spatial variations in the *within bog SS* of both natural and degraded bogs. Sampling at two natural bogs, Moyclare and Mount Hevey, in autumn 2021 recorded elevated BOD levels (27 and 19 mg O_2_/L respectively), which would suggest a physical stagnation of the bog streams at that time. However, samples taken downstream from natural bogs over the course of this study exhibited lower BOD than upstream samples and therefore were not a source of pollution. BOD concentrations were higher downstream from degraded sites. Mean values remained below the “high status” EQS (< 1.3 mg O_2_/L; SI 272 2009) in both groups, which suggests an adequate aeration effect beyond the bog environment. However, this may remain an issue at specific sites. For example we recorded values ranging from 3 to 9 mg O_2_/L at Corralanna and Ballybeg in downstream *RS,* thus requiring site-specific interventions as this would have several direct effects on the biodiversity of aquatic communities, as well as on the microbiological quality of these small streams (Donahue et al., 2022).

### Impacts on aquatic ecosystem services

Degraded water quality in this region is likely to be impacting on other aquatic ecosystem services, including biodiversity and microbial functioning (e.g. biogeochemical cycling). However, there has been limited work assessing the impacts of peat extraction associated degradation of water quality on aquatic communities in Irish streams. A recent review on drainage and extraction and associated water quality parameters (such as ammonia, sedimentation and heavy metals) found negative impacts on aquatic macro biota (Donahue et al., 2022). These included habitat alterations, reduced richness and altered community structure, as well as behavioural impacts and greater mortality. Such changes in aquatic ecosystem structure and functioning are likely to have knock-on impacts to the food webs and wider catchment/regional biodiversity and ecosystem services. However, it is thought these effects may be reversible following catchment scale peatland restoration (Ramchunder et al., 2012). While this work demonstrates less than ideal water quality for Irish midland streams and associations with peat extraction, more work is needed to also monitor associated aquatic communities, such as regional surveys or monitoring before and after peatland restoration.

### Towards a seasonal monitoring tool

While our results show large spatial and temporal variations associated with the wide range of site characterisations, they also revealed the baseline water quality in a landscape which is critical for future restoration work. While terrestrial biota of most protected bogs is carefully monitored in terms of habitat integrity (NPWS, 2017), there is often a lack of information regarding the aquatic environment associated with these ecosystems. Targets are often only concerned with terrestrial criteria (e.g. vegetation). Long-term monitoring of the chemical composition of bog water and associated waterbodies in conjunction with bog hydrological data is critical for a full understanding of the impacts of large-scale efforts to rewet/restore peatlands, as well as the development of robust eco-hydrological targets.

In Canada, where peatland restoration has been carried out for several decades, the analysis of water chemistry is viewed as an essential part of the monitoring of a restoration project, and is considered more informative than peat chemistry to assess the return of the natural balance of chemical elements in the bog itself (Andersen et al., 2010). In the UK, a comparative analysis of the costs and merits of different restoration methods showed that peatland restoration can result in significant recovery of hydrology and water quality within 10 years, while some deviation from intact hydrology is still typical (Artz et al., 2018). In contrast, Peacock et al. (2018) reported little impact of rewetting on DOM quality and, therefore, treatability of UK drinking water supplies, four years after ditch blocking in a degraded blanket bog.

Irish studies therefore must be financially supported to monitor the effect of such activities over long period. Restoration methods have only recently (< 10 years) been intensively deployed on the network of Irish SAC raised bog sites. It is expected that the benefits of restoration on water quality will only be evident in the medium-term (Menberu et al., 2017). While we have found lower DOC concentrations in *within bog SS*_nat_, de facto a useful monitoring indicator for bog restoration programme whose efforts is to reduce waterborne carbon losses (Evans et al., 2016b), high concentrations within all the receiving (up and below) streams demonstrate the complexity of this landscape that has suffered widespread disturbances over space and time. It may take several decades before the effects of bog restoration have any beneficial impact on the regional water quality (Evans et al., 2016a). However, our study provides at least a benchmark to evaluate the long-term performance of the rewetting and restoration activities that are being intensified in this region. A long-term monitoring programme of water chemistry is a critical supplementary tool towards the sustainable management of this peatland resource. To this effect, this study points to some basic components of such monitoring tool. First, EC values in our study consistently showed markedly greater values in degraded bogs (both *within bog SS* and *downstream RS* compared to natural sites). We also found a significant positive relationship between EC and TDS, Ca and TC values. Since EC measurement is inexpensive and rapid, widespread and routine monitoring could be deployed around bog sites to ascertain the status of the site but also monitor the effects of management actions (e.g. rewetting). Second, monitoring and modelling of DOC and TDN may be useful rewetting/restoration indicators, as well as informing global concerns for biodiversity and future climate effects (Edokpa et al., 2017b; Ritson et al., 2017; Kritzberg et al., 2020).

Finally, seasonal sampling may also impact water quality from these ecosystems and should, therefore, be carefully addressed in any monitoring programme. For example, our results indicate greater concentrations of various N species in the spring, as well as lower SUVA_254_ values (i.e. less labile DOM which are more difficult to treat). This is also correlated with high Fe in spring which is not bound to the higher aromatic hydrophilic NOM. Therefore, seasonal monitoring and especially spring measurements may be important in order to develop end-of-pipe treatment solutions in this region.

## Limitations and future research

This study presents water quality data collected over three sampling periods, encompassing two hydrological years and two seasons (autumn 2020, and spring and autumn of 2021). Water quality information is needed for full/multiple hydrological year(s) to truly understand the impacts of peatland degradation on water quality and to inform policy. A key difficulty of this study was the logistical issues that surround the sampling of a large number of sites and the timely laboratory analysis of a wide array of water quality parameters. In addition, the aromatic and coloured nature of bog water creates difficulties in laboratory analysis and some methods typically used for other waters (such as colorimetric) are not always possible. These issues and limitations should be considered when implementing future research/monitoring programmes.

With regard to the overall negative picture of water quality provided for the first time for this degraded bog landscape, this study recommends more specific investigations into the various drivers that affect water composition at the site- and landscape-level. Thus, we recommend that more efforts are deployed to obtain a continuous picture of both TDN and DOC concentrations within receiving streams rather than the current sporadic monitoring at EPA monitoring stations.

In addition, the characterisation of each bog site and associated bog streams should be part of a larger investigation into the effects of rewetting/restoration of extracted bogs, especially given the heterogeneity of each bog site and their associated bog streams. Of note, the historical drainage (several decades) and their associated long-term effects must also be taken into consideration in any expected timeline of response to new management practices and, therefore, adjust the duration of the monitoring period. The nutrients released from long-term degradation must also be considered as when they are combined with the neutral pH present in the drainage water (due to alkaline geology and the widespread presence of marl-bottom peat in this region), they provide optimal conditions for further peat mineralisation, leading to further challenges in terms of bog restoration projects (Konvalinková & Prach, 2014; Gaffney et al., 2018b). Altogether, such monitoring programmes would facilitate the adoption of timely, site-optimum management plans, as well as an associated land-use policy to facilitate the timely ecological recovery of this degraded landscape.

## Regulations and policy relevance for future utilisation of peatlands

There is a global recognition of the value of managing peatlands sustainably as a contribution to the implementation of many international agendas that include Sustainable Development, UNFCCC, Convention on Biological Diversity, Ramsar Convention, Sendai Framework for Disaster Risk Reduction 2030 and more targeted resolutions (e.g. Climate EU regulations, European Parliament, 2018). There is increasing evidence that peatland rewetting/restoration can be a tool to meet different regulatory targets, especially for the climate and biodiversity (Renou-Wilson et al., 2019; Wilson et al., 2022), but also in relation to WFD and its target to achieve ‘at least good status’ in European water bodies (Martin-Ortega et al., 2014) and to assist with drinking water treatments (O'Driscoll et al., 2018; Flynn et al., 2021). Ireland has the highest level of wetland loss (mostly peatlands) in the world since the 1700s (Fluet-Chouinard et al., 2023), and reversing centuries of unsustainable peatland management will require considerable efforts from all stakeholders. The full impacts of continued peat extraction must be acknowledged and properly assessed ahead of proposed land use, energy and horticultural projects which all require drainage. We recommend the deployment of a multi-pronged approach that focuses at both the site- and landscape-level.

Tackling the pollution within specific site bog streams will be largely addressed by (1) rewetting and rehabilitation projects within existing drained bogs where extraction has ceased, and (2) strict legal requirements for the continued licensing of peat extraction activities. Our results provide baseline information for future monitoring and set achievable targets. Where licensing activities are sought for continued peat extraction in Ireland, new guidance (albeit non-binding) have been established for the preparation of bog rehabilitation plans with licensees requiring (a) full characterisation of the bog, (b) extensive consultations with all stakeholders, (c) a test programme with list of criteria, and (d) an aftercare maintenance programme, before final surrender of the licence (EPA, 2020a). However, the water protection methods must be clearly validated in each specific environmental location in combination with specific regional objectives and additional methods tested to ensure improvement, along with seasonal, long-term monitoring.

At the landscape-level, the utilisation of the peat soils resource is to be integrated into cross-governmental policies to harmonise sustainable management of this large but diminishing resource. A holistic view of the management of peatlands is required, reflecting the importance of peatland functioning within a regional ecosystem. Our study is a timely overview of the status of water bodies during a period of transition in peatland management in this region. The Irish government has initiated plans for large-scale rewetting of its publicly-owned drained peatlands: more than 33,000 ha of industrial cutaway peatlands sites owned by Bord na Móna are targeted for rewetting (DECC, 2020) and a further 22,100 ha of raised bogs (both public and private) within the Natura 2000 network (Government of Ireland, 2021), while also promoting the rewetting of privately-owned drained peat soils (Government of Ireland, 2020). A suite of measures put in place to support communities and workers affected by the cessation of peat extraction has also been promoted by the Just Transition Fund. The current associated research that has mainly focused on water table and greenhouse gas fluxes must expand to include the monitoring of water quality in the whole region in order to help set optimal targets for the rewetting/restoration and subsequent maintenance programmes.

## Conclusion

This paper presented a snapshot of the quality of streams in the Irish midlands, a region known for its historical expanse of raised bogs, most of which have been affected by peat extraction. Here, we investigated within-bog streams and receiving streams associated with restored (near-natural) SAC raised bogs (all of which have undergone domestic peat extraction in the past and are here used as a proxy for natural sites) and compared them with those streams associated with industrially extracted cutaway peatlands (degraded sites). We confirmed our first hypothesis that within degraded bogs, small streams exhibited greater levels of pollutants, in particular: TDN and SO_4_, as well as higher EC and lower SUVA_254_. However, our second hypothesis was not confirmed as the chemical composition of streams that receive these bog waters did not significantly differ between near-natural and degraded sites; only at specific sites, were nitrogen pollutants attributed to the bog stream, reflecting the spatio-temporal scales of disturbance in this complex peat-scape. Our study points to required actions at both site-level (water treatment) and landscape-level (large-scale rewetting of drained bogs) to assist with meeting water quality standards in the region. To this effect, we support the long-term seasonal monitoring of the water chemistry of bog water and receiving streams (EC, DOC and TDN) as an important *indicator* to evaluate current and future peatland management activities in the region, especially rewetting/restoration efforts.

## Supplementary Information

Below is the link to the electronic supplementary material.

Supplementary file1 (DOCX 77 kb)

## Data Availability

The datasets generated during and/or analysed during the current study are available from the corresponding author on reasonable request.
