# Supplementary material for: An examination of the influence of drained peatlands on regional stream water chemistry
Source: Hydrobiologia. 2023 Mar 28;850(15):3313–39. doi: 10.1007/s10750-023-05188-5 (PMC10307720; doi:10.1007/s10750-023-05188-5)
Supplement: Supplementary file 1 — Supplementary file1 (DOCX 77 kb) [file 10750_2023_5188_MOESM1_ESM.docx]

**Supplementary material**

*Online Resource 1: Summary of the bogs study sites (see Fig. 1 for map), their status (SAC = Special Area of Conservation) together with sampled streams within bog small stream (SS), and upstream and downstream and receiving stream (RS), their code names, order, Water Framework Directive (WFD) sub-catchment area in km^2^ and sub-catchment/catchment name.*

| Bog Site | Site Status (year of restoration) | River name, order and WFD sub-catchment area in km^2^ | River bottom type | Sub-catchment and catchment name | Stream Type Sampled | | |
| --- | --- | --- | --- | --- | --- | --- | --- |
|  |  |  |  |  | Within-bog SS | Upstream RS | Downstream RS |
| Ardagullion, Co. Longford | Natural/restored SAC (2018) | Camlin 26, IE_SH_26C010050, order 1, 28 | Soft peaty | Camlin_SC_010,  26C Upper Shannon |  |  | ✓ |
| Bellanagare, Co. Roscommon | Natural/restored SAC (2020) | Francis, IE_SH_26F050050, order 1, 24 | Cobble/gravel | Suck_SC_010,  26D Upper Shannon | ✓ |  |  |
|  |  | IE_SH_26T030300, order 2, 24 | Cobble/gravel | Suck_SC_010,  26D Upper Shannon | ✓ |  |  |
|  |  | Trk_of_D, IE_SH_26C020100, order 1, 19 | Cobble/gravel | Breedoge_SC_010, 26B Upper Shannon | ✓ |  |  |
| Carrowbehy, Co. Roscommon | Natural/restored SAC (2019) | Cloonfower stream, IE_SH_26C210200, order 2, 19 | Cobble/gravel upstream  Peaty downstream | Suck_SC_010,  26D Upper Shannon |  | ✓ | ✓ |
|  |  | Gortaganwy stream, IE_SH_26C210200,  0rder 1, 19 | Cobble/gravel | Suck_SC_010,  26D Upper Shannon |  | ✓ |  |
| Carrownagappul, Co. Galway | Natural/restored SAC (1990 onwards and dams in 2019) | Gunnode stream, IE_SH_26S030100, order 2, 34 | Soft peaty | Castlegar_SC_010,  26D Upper Shannon | ✓ | ✓ | ✓ |
|  |  | Cloonconore stream, IE_SH_26S030100, order 1, 34 | Soft peaty | Castlegar_SC_010,  26D Upper Shannon | ✓ |  |  |
| Crosswood, Co. Westmeath | Natural/restored SAC (2005) | IE_SH_26S021800, order 1, 109 | Soft peaty | Shannon [Lower]_SC_010, 26G Lower Shannon | ✓ |  |  |
| Ferbane, Co. Offaly | Natural/restored SAC (2019) | Holy Well Clongawny,  IE_SH_25B270110,  order 1, 32 | Cobble/gravel | Shannon [Lower]_SC_030, 25B Lower Shannon |  |  | ✓✓ |
| Garriskill, Co. Westmeath | Natural SAC/Restored | IE_SH_26R030100, order 1, 41 | Cobble/gravel | Inny [Shannon]_SC_051,  26F Upper Shannon |  | ✓ |  |
|  |  | Riffey, IE_SH_26R030100,  order 3 , 41 | Soft peaty | Inny [Shannon]_SC_051,  26F Upper Shannon |  |  | ✓ |
| Killyconny, Co. Cavan | Natural/restored SAC (2006-2009) | Feegat stream, IE_EA_07M030700,  order 1, 21 | Soft peaty | Moynalty_SC_010,  07 Boyne |  |  | ✓ |
| Mount Hevey, Co. Westmeath | Natural/restored SAC (2005) | Ballyhaw, IE_EA_07B340940,  order 1, 18 | Soft peaty | Boyne_SC_040,  07 Boyne | ✓ |  |  |
|  |  | Aghamore IE_EA_07K010200, order 1, 17 | Cobble/gravel | Boyne_SC_030,  07 Boyne | ✓ |  |  |
| Moyclare, Co. Offaly | Natural/restored SAC (1995-2005 and again in 2019) | Holy Well Clonbonniff, IE_SH_25B270200,  order 1, 57.5 | Soft peaty | Shannon [Lower]_SC_030, 25B Lower Shannon  Shannon | ✓ |  |  |
|  |  | Lisdaly stream,  IE_SH_25B270200, order 1, 57.5 | Cobble/gravel | Shannon [Lower]_SC_030, 25B Lower Shannon  Shannon | ✓ |  |  |
|  |  | Moyclare Stream, IE_SH_25B270200, order 2, 57.5 | Cobble/gravel | Shannon [Lower]_SC_030, 25B Lower Shannon  Shannon | ✓ | ✓ | ✓ |
| Raheenmore, Co. Offaly | Natural/restored SAC (1990 and again in 2020) | Bunsallagh, IE_EA_07Y020070, order 1, 29 | Soft peaty | Yellow [Castlejordan]_SC_010,  07 Boyne |  | ✓ | ✓ |
|  |  | Puttaghan 25 IE_SH_25S030100, order 1, 23 | Cobble gravel | Silver[Tullamore]_SC_010 | ✓ |  |  |
| All Saints Bog, Co. Offaly | Degraded/peat extraction | Rapemills, IE_SH_25R010500,  order 2, 36 | Cobble gravel upstream  Soft peaty downstream | Shannon [lower] 25B,  25B Lower Shannon | ✓✓ | ✓ | ✓ |
| Ballybeg/Togher, Co. Westmeath | Degraded/peat extraction | Coolcor stream, IE_EA_07C080190, order 1 upstream and 2 downstream | Cobble gravel upstream  Soft peaty downstream | Yellow [Castlejordan]_SC_010,  07 Boyne |  | ✓ | ✓ |
|  |  | Derryiron,  IE_EA_07Y020100,  Order 1, 13 | Soft peaty | Yellow [Castlejordan]_SC_010,  07 Boyne | ✓ |  |  |
| Ballydaly, Co. Offaly | Degraded/peat extraction | Kilcolgan beg,  IE_SH_25B090800,  order 1, 15 | Soft peaty | BROSNA_SC_060, 25A Lower Shannon | ✓ |  |  |
| Ballynaskeagh, Co. Westmeath | Degraded/peat extraction | Graffasntown, IE_EA_07D010200, order 1, 25 | Soft peaty | Deel [Raharney]_SC_010, 07 Boyne | ✓ |  |  |
| Coolcraff, Co. Westmeath | Degraded/peat extraction | Inny River, IE_SH_26I010700, order 4, 70 | Cobble/gravel upstream  Soft peaty downstream | Inny [Shannon] 26I01,  26E Upper Shannon | ✓ | ✓ | ✓ |
| Corralanna, Co. Westmeath | Degraded/peat extraction | Ferskill, IE_SH_26F370890, order 3, 26 | Cobble/gravel upstream  Soft peaty downstream | Inny [Shannon]_SC_019,  26F Upper Shannon | ✓ | ✓ | ✓ |
|  |  | Coolnagun stream, IE_SH_26C080860, order 1 upstream, order 2 downstream, 15 | Soft peaty upstream and downstream | Inny [Shannon]_SC_020,  26F Upper Shannon | ✓✓ | ✓ | ✓ |
| Derrybrat, Co. Offaly | Degraded/peat extraction | Silver, IE_SH_25S020701, order 4, 16 | Soft peaty | Brosna_SC_070,  25A Lower Shannon | ✓ ✓ | ✓ | ✓ |
| Derrycarney, Co. Offaly | Degraded/peat extraction | Derrycarney, IE_SH_25B090950, order 1, 39 | Soft peaty | BROSNA_SC_070, 25A Lower Shannon | ✓ |  |  |
| Derrycooly, Co. Offaly | Degraded/peat extraction | Derrycooly stream, IE_SH_25P050300, order 1, 35 | Soft peaty | Brosna_SC_050, 25A Lower Shannon | ✓ |  |  |
| Drinagh | Degraded/peat extraction | Derrymullin and Loughderry River, IE_SH_25L010090, order 2, 11 | Soft peaty with some mineral sediments | Brosna_SC_070,  25 A Lower Shannon |  | ✓ |  |
|  |  | Little [Cloghan], IE_SH_25L01009,  order 3, 20 | Soft peaty | Brosna_SC_070,  25 A Lower Shannon | ✓ |  | ✓ |
| Falsk, Co. Offaly | Degraded/peat extraction | Falsk 25, IE_SH_25B090950, order 1, 39 | Soft peaty | Brosna_SC_070, 25A Lower Shannon | ✓ |  |  |
| Rossan, Co. Meath | Degraded/peat extraction | Rossan 07, IE_EA_07K010100,  Order 1, 18 | Soft peaty | Boyne_SC_030, 07 Boyne | ✓ |  |  |

*Online Resource 2: Summary of ANOVA statistical tests run for each hypothesis, including factors and levels used in ANOVA models. ‘n’ represents the sample number.*

| Hypothesis | Test | Factors | Levels | *n* |
| --- | --- | --- | --- | --- |
| #1 Water quality parameters of *within bog* *SS* are significantly different between sampling periods. | ANOVA 1a | Sampling time | Autumn 2020 Spring 2021 Autumn 2021 | 6  27  22 |
| #2 Water quality parameters of *RS* are significantly different between sampling periods. | ANOVA 1b | Sampling time | Autumn 2020 Spring 2021 Autumn 2021 | 8  32  32 |
| #3 Water quality parameters are significantly different in *within bog SS* in degraded bogs compared to natural bogs. | ANOVA 2 | Site status | Degraded  Natural | 35  20 |
|  |  | Sampling time | Autumn 2020 Spring 2021 Autumn 2021 | 6  27  22 |
| #4 Water quality parameters are significantly different downstream compared to upstream of degraded bogs. | ANOVA 3a | Sampling location | Upstream RS  Downstream RS | 20  18 |
|  |  | Sampling time | Autumn 2020 Spring 2021 Autumn 2021 | 7  15  16 |
| #5 Water quality parameters are significantly different upstream compared to downstream of natural bogs. | ANOVA 3b | Sampling location | Upstream RS  Downstream RS | 12  22 |
|  |  | Sampling time | Autumn 2020 Spring 2021 Autumn 2021 | 1  17  16 |
| #6 Water quality parameters are significantly different upstream and downstream of specific bogs. | ANOVA 4 | Sites | 10 bogs | 2-10 per bog |
|  |  | Sampling location | Upstream Downstream | 26  24 |
|  |  | Sampling time | Autumn 2020 Spring 2021 Autumn 2021 | 5  23  22 |

*Online Resource 3: Results of ANOVA 1, and transformations applied to data prior to analysis if required. Bold p values were deemed significant (p = <0.05).*

| Parameter | Sampling Time | F Value | Degrees of Freedom | Transformation |
| --- | --- | --- | --- | --- |
| pH | **<0.001** | 9.304 | 2 | None |
| Conductivity | **<0.001** | 10.161 | 2 | None |
| Turbidity | 1.000 | 0.647 | 2 | Log |
| DOC | 1.000 | 1.669 | 2 | None |
| SUVA_254_ | **0.048** | 9.977 | 1 | None |
| TDN | **<0.001** | 9.688 | 2 | None |
| TAN | 1.000 | 0.854 | 2 | Sqrt |
| NO_3_-N | **<0.001** | 12.618 | 1 | None |
| NO_2_-N | 1.000 | 0.335 | 2 | Sqrt |
| TDP | 1.000 | 0.968 | 2 | None |
| OP | 1.000 | 2.361 | 2 | None |
| SO_4_ | 0.144 | 5.028 | 2 | Log |
| Na | 1.000 | 0.497 | 1 | Log |
| K | 1.000 | 2.105 | 1 | None |
| Fe | **<0.001** | 9.396 | 2 | Sqrt |
| BOD | 1.000 | 0.508 | 2 | None |

*Online Resource 4: Results of ANOVA 2, and transformations applied to data prior to analysis if required. Bold p values were deemed significant (p = <0.05).*

| Parameter | Anova Result | Site Status | Sampling Time | Site Status: Sampling Time | Transformation |
| --- | --- | --- | --- | --- | --- |
| pH | p | 1.000 | 0.240 | 1.000 | None |
|  | F | 3.622 | 5.800 | 2.397 |  |
|  | Df | 1 | 2 | 1 |  |
| Conductivity | p | **<0.001** | 1.000 | 1.000 | None |
|  | F | 12.753 | 3.844 | 0.005 |  |
|  | Df | 1 | **2** | 1 |  |
| Turbidity | p | 1.000 | 1.000 | 1.000 | Log |
|  | F | 0.205 | 3.591 | 0.504 |  |
|  | Df | 1 | 2 | 1 |  |
| DOC | p | 1.000 | 1.000 | 1.000 | None |
|  | F | 3.832 | 0.061 | 3.983 |  |
|  | Df | 1 | 2 | 1 |  |
| SUVA_254_ | p | **<0.001** | **<0.001** | 0.384 | None |
|  | F | 15.157 | 21.643 | 7.611 |  |
|  | Df | 1 | 1 | 1 |  |
| TDN | p | **0.048** | 1.000 | 1.000 | None |
|  | F | 11.471 | 0.029 | 0.002 |  |
|  | Df | 1 | 2 | 1 |  |
| TAN | p | 1.000 | 1.000 | 1.000 | Sqrt |
|  | F | 1.620 | 0.625 | 0.545 |  |
|  | Df | 1 | 2 | 1 |  |
| NO_3_-N | p | 1.000 | 1.000 | 1.000 | Sqrt |
|  | F | 0.637 | 0.026 | 0.006 |  |
|  | Df | 1 | 1 | 1 |  |
| NO_2_-N | p | 1.000 | 1.000 | 1.000 | Sqrt |
|  | F | 1.208 | 2.456 | 1.149 |  |
|  | Df | 1 | 2 | 1 |  |
| TDP | p | 1.000 | 1.000 | 1.000 | Sqrt |
|  | F | 0.095 | 0.368 | 1.050 |  |
|  | Df | 1 | 2 | 1 |  |
| OP | p | 1.000 | 1.000 | 1.000 | None |
|  | F | 1.298 | 1.457 | 1.087 |  |
|  | Df | 1 | 2 | 1 |  |
| SO_4_ | p | **<0.001** | 1.000 | 1.000 | Sqrt |
|  | F | 13.741 | 3.260 | 1.474 |  |
|  | Df | 1 | 2 | 1 |  |
| Na | p | 1.000 | 1.000 | 1.000 | Sqrt |
|  | F | 0.994 | 0.286 | 0.367 |  |
|  | Df | 1 | 1 | 1 |  |
| K | p | 1.000 | 1.000 | 1.000 | Sqrt |
|  | F | 2.284 | 0.316 | 1.565 |  |
|  | Df | 1 | 1 | 1 |  |
| Fe | p | 1.000 | **<0.001** | 1.000 | Log |
|  | F | 9.438 | 8.175 | 1.057 |  |
|  | Df | 1 | 2 | 1 |  |
| BOD | p | 1.000 | 1.000 | 1.000 | Sqrt |
|  | F | 0.837 | 2.820 | 3.773 |  |
|  | Df | 1 | 2 | 1 |  |

*Online Resource 5: Results of ANOVA 3a, and transformations applied to data prior to analysis if required. No p values were deemed significant (p = <0.05).*

| Parameter | Anova Result | Site Status | Sampling Time | Site Status: Sampling Time | Transformation |
| --- | --- | --- | --- | --- | --- |
| pH | p | 1.000 | 0.144 | 1.000 | None |
|  | F | 0.101 | 7.236 | 1.417 |  |
|  | Df | 1 | 2 | 2 |  |
| Conductivity | p | 1.000 | 0.768 | 1.000 | None |
|  | F | 0.229 | 4.722 | 2.243 |  |
|  | Df | 1 | 2 | 2 |  |
| Turbidity | p | 1.000 | 1.000 | 1.000 | Log |
|  | F | 2.869 | 1.903 | 0.033 |  |
|  | Df | 1 | 2 | 2 |  |
| DOC | p | 1.000 | 1.000 | 1.000 | None |
|  | F | 0.099 | 3.041 | 1.156 |  |
|  | Df | 1 | 2 | 2 |  |
| SUVA_254_ | p | 1.000 | 0.672 | 1.000 | Log |
|  | F | 0.164 | 6.838 | 0.046 |  |
|  | Df | 1 | 1 | 1 |  |
| TDN | p | 1.000 | 1.000 | 1.000 | None |
|  | F | 0.578 | 4.159 | 0.623 |  |
|  | Df | 1 | 2 | 2 |  |
| TAN | p | 1.000 | 1.000 | 1.000 | Sqrt |
|  | F | 0.544 | 0.197 | 1.029 |  |
|  | Df | 1 | 2 | 2 |  |
| NO_3_-N | p | 1.000 | 0.384 | 1.000 | Log |
|  | F | 1.910 | 8.085 | 0.414 |  |
|  | Df | 1 | 1 | 1 |  |
| NO_2_-N | p | 1.000 | 1.000 | 1.000 | Sqrt |
|  | F | 2.504 | 1.524 | 0.636 |  |
|  | Df | 1 | 2 | 2 |  |
| TDP | p | 1.000 | 1.000 | 1.000 | None |
|  | F | 3.021 | 1.822 | 2.090 |  |
|  | Df | 1 | 2 | 2 |  |
| OP | p | 1.000 | 0.816 | 0.624 | Sqrt |
|  | F | 0.650 | 4.647 | 5.004 |  |
|  | Df | 1 | 2 | 2 |  |
| SO_4_ | p | 1.000 | 1.000 | 1.000 | Log |
|  | F | 5.338 | 2.481 | 1.773 |  |
|  | Df | 1 | 2 | 2 |  |
| Na | p | 1.000 | 1.000 | 1.000 | None |
|  | F | 0.004 | 0.137 | 0.014 |  |
|  | Df | 1 | 1 | 1 |  |
| K | p | 1.000 | 1.000 | 1.000 | None |
|  | F | 0.208 | 0.408 | 0.360 |  |
|  | Df | 1 | 1 | 1 |  |
| Fe | p | 1.000 | 0.096 | 1.000 | None |
|  | F | 0.052 | 7.366 | 0.254 |  |
|  | Df | 1 | 2 | 2 |  |
| BOD | p | 1.000 | 1.000 | 1.000 | None |
|  | F | 0.006 | 0.350 | 0.656 |  |
|  | Df | 1 | 2 | 2 |  |

*Online Resource 6: Results of ANOVA 3b, and transformations applied to data prior to analysis if required. Bold p values were deemed significant (p = <0.05).*

| Parameter | Anova Result | Site Status | Sampling Time | Site Status: Sampling Time | Transformation |
| --- | --- | --- | --- | --- | --- |
| pH | p | 1.000 | 0.192 | 1.000 | None |
|  | F | 0.679 | 6.814 | 0.108 |  |
|  | Df | 1 | 2 | 1 |  |
| Conductivity | p | 1.000 | **0.048** | 1.000 | None |
|  | F | 1.021 | 8.639 | 0.137 |  |
|  | Df | 1 | 2 | 1 |  |
| Turbidity | p | 1.000 | 1.000 | 1.000 | Log |
|  | F | 0.085 | 0.153 | 0.783 |  |
|  | Df | 1 | 2 | 1 |  |
| DOC | p | 1.000 | 1.000 | 1.000 | None |
|  | F | 0.650 | 0.802 | 1.310 |  |
|  | Df | 1 | 2 | 1 |  |
| SUVA_254_ | p | 0.384 | 1.000 | 1.000 | None |
|  | F | 8.047 | 5.708 | 0.298 |  |
|  | Df | 1 | 1 | 1 |  |
| TDN | p | 1.000 | 0.192 | 1.000 | None |
|  | F | 0.866 | 6.811 | 1.478 |  |
|  | Df | 1 | 2 | 1 |  |
| TAN | p | 1.000 | 1.000 | 1.000 | None |
|  | F | 0.028 | 1.895 | 0.066 |  |
|  | Df | 1 | 2 | 1 |  |
| NO_3_-N | p | 1.000 | 0.144 | 1.000 | None |
|  | F | 0.399 | 10.732 | 0.043 |  |
|  | Df | 1 | 1 | 1 |  |
| NO_2_-N | p | 1.000 | 0.912 | 1.000 | None |
|  | F | 0.170 | 4.572 | 0.631 |  |
|  | Df | 1 | 2 | 1 |  |
| TDP | p | 1.000 | 1.000 | 1.000 | None |
|  | F | 3.390 | 0.204 | 2.342 |  |
|  | Df | 1 | 2 | 1 |  |
| OP | p | 1.000 | 1.000 | 1.000 | None |
|  | F | 4.101 | 0.009 | 0.357 |  |
|  | Df | 1 | 2 | 1 |  |
| SO_4_ | p | 1.000 | 0.672 | 1.000 | None |
|  | F | 0.119 | 4.954 | 0.004 |  |
|  | Df | 1 | 2 | 1 |  |
| Na | p | 1.000 | 1.000 | 1.000 | None |
|  | F | 0.653 | 0.012 | 0.001 |  |
|  | Df | 1 | 1 | 1 |  |
| K | p | 1.000 | 1.000 | 1.000 | Log |
|  | F | 2.210 | 1.637 | 1.246 |  |
|  | Df | 1 | 1 | 1 |  |
| Fe | p | 1.000 | 0.240 | 1.000 | Log |
|  | F | 0.002 | 6.342 | 0.104 |  |
|  | Df | 1 | 2 | 1 |  |
| BOD | p | 1.000 | 1.000 | 1.000 | None |
|  | F | 0.399 | 0.123 | 0.019 |  |
|  | Df | 1 | 2 | 1 |  |

*Online Resource 7: Results of ANOVA 4, and transformations applied to data prior to analysis if required. Bold p values are deemed significant (p = <0.05). Posthoc interactions of interest are shown where the interaction between site and sampling location (upstream vs. downstream) was significant.*

| Parameter | Anova Result | Site | Sampling Location | Sampling Time | Site: Sampling Location | Site: Sampling Time | Sampling Location: Sampling Time | Site: Sampling Location: Sampling Time | Transformation | Posthoc result |
| --- | --- | --- | --- | --- | --- | --- | --- | --- | --- | --- |
| pH | p | 1.000 | 1.000 | 0.448 | 1.000 | 1.000 | 1.000 | 1.000 | None |  |
|  | F | 5.724 | 0.968 | 16.778 | 1.095 | 1.699 | 0.225 | 0.685 |  |  |
|  | Df | 9 | 1 | 2 | 9 | 11 | 2 | 9 |  |  |
| Conductivity | p | **0.000** | 1.000 | **0.000** | 1.000 | 1.000 | 1.000 | 1.000 | None |  |
|  | F | 31.925 | 0.027 | 37.230 | 1.678 | 3.828 | 0.888 | 0.740 |  |  |
|  | Df | 9 | 1 | 2 | 9 | 11 | 2 | 9 |  |  |
| Turbidity | p | 1.000 | 1.000 | 1.000 | 1.000 | 1.000 | 1.000 | 0.784 | None |  |
|  | F | 7.859 | 0.041 | 5.521 | 8.233 | 3.105 | 2.449 | 8.997 |  |  |
|  | Df | 9 | 1 | 2 | 9 | 11 | 2 | 9 |  |  |
| DOC | p | 1.000 | 1.000 | 1.000 | 1.000 | 1.000 | 1.000 | 1.000 | None |  |
|  | F | 1.660 | 0.000 | 2.215 | 0.262 | 1.635 | 0.623 | 0.911 |  |  |
|  | Df | 9 | 1 | 2 | 9 | 11 | 2 | 9 |  |  |
| SUVA_254_ | p | **0.000** | 1.000 | 0.112 | 1.000 | 1.000 | 1.000 | 1.000 | None |  |
|  | F | 36.758 | 0.725 | 44.172 | 1.730 | 7.409 | 0.621 | 0.747 |  |  |
|  | Df | 9 | 1 | 1 | 9 | 9 | 1 | 8 |  |  |
| TDN | p | 0.672 | 1.000 | 1.000 | 1.000 | 1.000 | 1.000 | 1.000 | None |  |
|  | F | 10.036 | 0.805 | 10.830 | 0.446 | 1.114 | 0.757 | 0.406 |  |  |
|  | Df | 9 | 1 | 2 | 9 | 11 | 2 | 9 |  |  |
| TAN | p | 0.560 | 1.000 | 1.000 | 1.000 | 1.000 | 1.000 | 1.000 | None |  |
|  | F | 10.205 | 1.269 | 0.688 | 3.717 | 0.536 | 2.236 | 2.140 |  |  |
|  | Df | 9 | 1 | 2 | 9 | 11 | 2 | 9 |  |  |
| NO_3_-N | p | **0.000** | 1.000 | **0.000** | 1.000 | 0.224 | 1.000 | 1.000 | None |  |
|  | F | 76.123 | 1.532 | 183.297 | 3.900 | 13.574 | 1.750 | 1.876 |  |  |
|  | Df | 9 | 1 | 2 | 9 | 11 | 2 | 9 |  |  |
| NO_2_-N | p | 0.112 | 0.560 | 0.560 | 0.336 | 1.000 | 1.000 | 1.000 | None |  |
|  | F | 19.318 | 19.404 | 14.436 | 12.535 | 6.482 | 1.906 | 3.493 |  |  |
|  | Df | 9 | 1 | 2 | 9 | 11 | 2 | 9 |  |  |
| TDP | p | 1.000 | 1.000 | 1.000 | 1.000 | 1.000 | 1.000 | 1.000 | None |  |
|  | F | 3.556 | 0.183 | 0.255 | 0.945 | 0.669 | 0.140 | 1.113 |  |  |
|  | Df | 9 | 1 | 2 | 9 | 11 | 2 | 9 |  |  |
| OP | p | 0.336 | **0.000** | **0.000** | 0.336 | 0.224 | **0.000** | 0.672 | None |  |
|  | F | 8.501 | 35.354 | 35.178 | 8.482 | 8.488 | 48.884 | 3.077 |  |  |
|  | Df | 9 | 1 | 2 | 9 | 10 | 2 | 8 |  |  |
| SO_4_ | p | **0.000** | **0.000** | **0.000** | **0.000** | 0.672 | 0.672 | 0.448 | Sqrt | Drinagh p = <0.001  Ballybeg p = <0.001 |
|  | F | 211.454 | 160.690 | 115.350 | 86.076 | 9.384 | 13.535 | 11.358 |  |  |
|  | Df | 9 | 1 | 2 | 9 | 11 | 2 | 9 |  |  |
| Na | p | 1.000 | 1.000 | 1.000 | 1.000 | 1.000 | 1.000 | 1.000 | Sqrt |  |
|  | F | 3.516 | 0.057 | 0.821 | 0.190 | 0.283 | 0.099 | 0.253 |  |  |
|  | Df | 9 | 1 | 1 | 9 | 9 | 1 | 8 |  |  |
| K | p | **0.000** | 0.672 | **0.000** | 0.784 | 0.112 | 1.000 | 0.224 | None |  |
|  | F | 76.489 | 17.586 | 194.674 | 9.406 | 24.804 | 9.723 | 13.817 |  |  |
|  | Df | 9 | 1 | 1 | 9 | 9 | 1 | 8 |  |  |
| Fe | p | **0.000** | 1.000 | **0.000** | 0.112 | 1.000 | 1.000 | 0.224 | None |  |
|  | F | 36.514 | 7.008 | 50.235 | 17.103 | 6.953 | 9.863 | 14.024 |  |  |
|  | Df | 9 | 1 | 2 | 9 | 11 | 2 | 9 |  |  |
| BOD | p | 1.000 | 1.000 | 1.000 | 1.000 | 1.000 | 1.000 | 1.000 | Sqrt |  |
|  | F | 1.418 | 0.553 | 0.159 | 1.131 | 0.665 | 0.824 | 0.847 |  |  |
|  | Df | 9 | 1 | 2 | 9 | 11 | 2 | 9 |  |  |

*Online Resource 8: Loading values from PCA analysis. PCA1 compared within bog SSnat and within bog SSdeg water quality, and PCA2 compared the water quality of downstream RS (from degraded and natural bogs). Higher values > ± 0.30 are in bold.*

| Parameter | PCA1 | | PCA2 | |
| --- | --- | --- | --- | --- |
|  | PC1 | PC2 | PC1 | PC2 |
| BOD | -0.01 | 0.23 | 0.03 | **0.32** |
| Ca | **-0.31** | 0.03 | -0.18 | 0.22 |
| Cl | -0.23 | -0.27 | -0.23 | -0.08 |
| DIN | -0.23 | **0.30** | **-0.30** | 0.05 |
| DOC | 0.02 | -0.17 | -0.07 | -0.28 |
| EC | **-0.32** | -0.02 | **-0.35** | 0.09 |
| Fe | 0.07 | 0.04 | 0.18 | 0.11 |
| K | -0.28 | 0.01 | -0.12 | -0.02 |
| Mg | **-0.31** | -0.08 | -0.06 | 0.16 |
| Na | -0.21 | -0.26 | -0.14 | -0.03 |
| NH_3_-N | -0.13 | **0.37** | -0.09 | 0.21 |
| NO_2_-N | -0.15 | -0.06 | -0.09 | 0.24 |
| NO_3_-N | -0.25 | -0.03 | **-0.31** | -0.01 |
| OP | -0.02 | 0.13 | -0.02 | -0.04 |
| pH | -0.19 | -0.18 | -0.28 | -0.10 |
| SO4 | -0.17 | -0.22 | -0.07 | **0.30** |
| SUVA_254_ | 0.18 | 0.17 | 0.26 | 0.26 |
| TAN | -0.05 | **0.50** | 0.04 | **0.44** |
| TC | -0.27 | 0.08 | -0.23 | 0.06 |
| TDS | **-0.30** | -0.04 | **-0.35** | 0.09 |
| TIC | -0.28 | 0.20 | -0.21 | 0.14 |
| TDN | -0.19 | 0.18 | **-0.34** | 0.04 |
| TDP | -0.03 | 0.19 | 0.06 | **0.39** |
| Turbidity | -0.01 | 0.22 | 0.10 | 0.25 |

*Online Resource 9: Detection limits for water quality parameters analysed.*

| Water quality parameter | Abbreviation | Detection Limit |
| --- | --- | --- |
| Total carbon, total inorganic carbon, dissolved organic carbon | TC, TIC, DOC | 0.5 ug/L |
| Total dissolved nitrogen | TDN | 5 ug/L |
| Chloride (Cl^-^) | Cl | 0.25 mg/L |
| Nitrate (NO_3_^—^N) | NO_3_-N | 0.10 mg/L |
| Sulphate (SO_4_^2-^) | SO_4_ | 0.48 mg/L |
| Sodium (Na^+^) | Na | 0.01 mg/L |
| Magnesium (Mg^2+^) | Mg | 0.03 mg/L |
| Calcium (Ca^2+^) | Ca | 0.36 mg/L |
| Iron | Fe | 7.2 ug/L |
| Potassium | K | 0.05 mg/L |
| Nitrite | NO_2_-N | 0.005 mg N/L |
| Total Ammonia (TAN as N) | TAN | 0.01 mg N/L |
| Orthophosphate | OP | 0.025 mg/L |
| Turbidity | Turbidity | 0.5 NTU |
| Biological oxygen demand | BOD | 2 mg O_2_/L |
| Total dissolved phosphorus | TDP | 0.05 mg/L |
